# Unequal Recombination and Evolution of the Mating-Type (*MAT*) Loci in the Pathogenic Fungus *Grosmannia clavigera* and Relatives

**DOI:** 10.1534/g3.112.004986

**Published:** 2013-03-01

**Authors:** Clement K.-M. Tsui, Scott DiGuistini, Ye Wang, Nicolas Feau, Braham Dhillon, Jörg Bohlmann, Richard C. Hamelin

**Affiliations:** * Department of Forest and Conservation Sciences, The University of British Columbia, Vancouver, BC, Canada V6T 1Z4; † Department of Wood Science, The University of British Columbia, Vancouver, BC, Canada V6T 1Z4; ‡ Michael Smith Laboratory, The University of British Columbia, Vancouver, BC, Canada V6T 1Z4; § Natural Resources Canada, Laurentian Forestry Centre, Québec, QC, Canada G1V 4C7

**Keywords:** heterothallism, homothallism, mating system evolution, outcrossing, selfing

## Abstract

Sexual reproduction in fungi is regulated by the mating-type (*MAT*) locus where recombination is suppressed. We investigated the evolution of *MAT* loci in eight fungal species belonging to *Grosmannia* and *Ophiostoma* (Sordariomycetes, Ascomycota) that include conifer pathogens and beetle symbionts. The *MAT1-2* idiomorph/allele was identified from the assembled and annotated *Grosmannia clavigera* genome, and the *MAT* locus is flanked by genes coding for cytoskeleton protein (*SLA*) and DNA lyase. The synteny of these genes is conserved and consistent with other members in Ascomycota. Using sequences from *SLA* and flanking regions, we characterized the *MAT1-1* idiomorph from other isolates of *G. clavigera* and performed dotplot analysis between the two idiomorphs. Unexpectedly, the *MAT1-2* idiomorph contains a truncated *MAT1-1-1* gene upstream of the *MAT1-2-1* gene that bears the high-mobility-group domain. The nucleotide and amino acid sequence of the truncated *MAT1-1-1* gene is similar to its homologous copy in the *MAT1-1* idiomorph in the opposite mating-type isolate, except that positive selection is acting on the truncated gene and the alpha(α)-box that encodes the transcription factor has been deleted. The *MAT* idiomorphs sharing identical gene organization were present in seven additional species in the Ophiostomatales, suggesting that the presence of truncated *MAT1-1-1* gene is a general pattern in this order. We propose that an ancient unequal recombination event resulted in the ancestral *MAT1-1-1* gene integrated into the *MAT1-2* idiomorph and surviving as the truncated *MAT1-1-1* genes. The α-box domain of *MAT1-1-1* gene, located at the same *MAT* locus adjacent to the *MAT1-2-1* gene, could have been removed by deletion after recombination due to mating signal interference. Our data confirmed a 1:1 MAT/sex ratio in two pathogen populations, and showed that all members of the Ophiostomatales studied here including those that were previously deemed asexual have the potential to reproduce sexually. This ability can potentially increase genetic variability and can enhance fitness in new, ecological niches.

Sexual reproduction in fungi is controlled by the mating-type (*MAT*) loci, in which the genes determine mating incompatibility and regulate key mating processes (Coppin *et al.* 1997; Debuchy *et al.* 2010). Most haploid ascomycete fungi have a single *MAT* locus termed *MAT1*, which has two alleles called *MAT1-1* and *MAT1-2* (Turgeon and Yoder 2000). Like large portions of the human X and Y chromosomes, the DNA and amino acid sequences of *MAT1-1* and *MAT1-2* are no more similar than expected by chance; thus, they have been termed idiomorphs (Metzenberg and Glass 1990). The idiomorphs encode transcription factors with conserved DNA binding domains involved in the regulation of mating identity and sexual development (Coppin *et al.* 1997). The two idiomorphs are distinguished by the presence of either an alpha(α)-box domain in *MAT1-1* or a high-mobility-group (*HMG*) domain in *MAT1-2* (Coppin *et al.* 1997). Heterothallic (outcrossing) ascomycete fungi carry either one of the two idiomorphs at the *MAT* locus, and two isolates bearing complementary *MAT1* idiomorphs are required to mate. In contrast, homothallic ascomycetes can undergo haploid selfing (completion of the sexual cycle with a clonemate) because *MAT* genes with α-box and HMG domains are both present in the mating partners (Debuchy *et al.* 2010; Billiard *et al.* 2011). This gene arrangement may have evolved under selection for universal compatibility (Billiard *et al.* 2011, 2012). The *MAT1-1* idiomorph usually comprises a gene, *MAT1-1-1*, whereas the *MAT1-2* idiomorph has a different gene, *MAT1-2-1*. However, additional genes such as *MAT1-1-2* and *MAT1-1-3* have been reported in the *MAT1-1* idiomorphs in various sordariomycetous ascomycetes (Turgeon and Yoder 2000; Debuchy *et al.* 2010). The questions of which mode of sexual reproduction, heterothallism or homothallism, is ancestral and which genetic/evolutionary mechanism mediates the change from one to the other have received much attention from fungal biologists (Debuchy *et al.* 2010). Therefore, understanding the *MAT* locus organization can provide insight into the genetics and evolution of mating systems and life history in ascomycetes.

The fungi in Ophiostomatales (Sordariomycetes, Ascomycota) are diverse, with more than 100 species, including many important or aggressive tree pathogens responsible for wilt diseases, blue stain in commercial timber; in addition, they play an important role as insect symbionts and associates, as well as saprophytes (Zipfel *et al.* 2006). Most species belong to sexual genera *Grosmannia*, *Ophiostoma*, and the asexual genus *Leptographium*. In North America, *Grosmannia clavigera* is an important conifer pathogen that has a symbiotic relationship with its vector the mountain pine beetle (*Dendroctonus ponderosae*). *Grosmannia clavigera* and other fungal pathogens and symbionts, such as *Leptographium longiclavatum* and *Ophiostoma montium*, are carried in the mycangia and on the exoskeleton of mountain pine beetles because these fungi produce abundant slimy spores that attach themselves to the insect’s body. These fungi can grow into the sapwood and damage the host tree’s water transport system (Yamaoka *et al.* 1995; Solheim and Krokene 1998). The mountain pine beetles and its fungal symbionts have destroyed more than 17.5 million ha of lodgepole pine forests in western Canada in the last decade (http://www.for.gov.bc.ca/hfp/mountain_pine_beetle/facts.htm), and the magnitude of devastation is the largest in recorded history in Canada (Kurz *et al.* 2008; Safranyik *et al.* 2010).

*G. clavigera* is very aggressive and it may be capable of detoxifying terpenoids that are an important class of defense compounds in pines (DiGuistini *et al.* 2011). Because *G. clavigera* plays an important role in the pine−beetle−fungus dynamics and epidemics, it is essential to understand its biology, genetics, and population structure. Populations of *G. clavigera* are polymorphic and form distinct genetic clusters yet with some gene flow and admixture among clusters (Lee *et al.* 2007; Tsui *et al.* 2012). Evidence of random mating and linkage equilibrium were also revealed, indicating sexual reproduction occurred in *G. clavigera* populations (Tsui *et al.* 2012). However, the sexual fruiting bodies of *G. clavigera* are rarely observed (Lee *et al.* 2005), even though *G. clavigera* is reported to have a life cycle comprising both the asexual and sexual stages. It is important to understand the ability of a fungal pathogen to perform sexual reproduction because such information could provide clues to the population biology of these fungi, which could be useful to further our understanding of the recent unprecedented epidemic.

*Grosmannia clavigera* lineage Gs *sensu* (Alamouti *et al.* 2011) is heterothallic because a gene homologous to *MAT1-2-1* is characterized at the *MAT* locus in the isolate for which the genome was sequenced but no gene coding for the α-box domain was reported (DiGuistini *et al.* 2011). Although fungi in Ophiostomatales are diverse in breeding strategies with worldwide distribution, the *MAT* locus has been characterized in only a limited number of species, such as *Ophiostoma ulmi*, *O. novo-ulmi*, and *O. himal-ulmi*, which are responsible for the Dutch elm disease epidemic in Europe and North America (Paoletti *et al.* 2005a; Jacobi *et al.* 2010). The genes corresponding to opposite mating-types recently were reported in the *MAT* locus of *Ophiostoma quercus*, which causes significant sapstain in hardwood (Wilken *et al.* 2012). Because the *MAT1-1* idiomorph has not been characterized in *G. clavigera* and the *MAT* locus organization has not been well investigated in other fungi of Ophiostomatales, we used genomics and primer walking approaches to study the organization and evolution of the *MAT* locus in *G. clavigera* and eight related fungi. To address the question of whether heterothallism or homothallism is a derived character state, we also compared the *MAT* locus organization with other ascomycete species in the Sordariomycetes. The aim of the present investigation was (1) to characterize the mating-type locus organization of *G. clavigera* bearing a *MAT1-1* idiomorph; (2) to investigate the evolution of *MAT* genes and mating systems in fungi belonging to Ophiostomatales with respect to other ascomycetes; and (3) to determine the mating-type ratio in the populations of *G. clavigera*.

## Materials and Methods

### Fungal materials and culture collection

Thirty-four fungal isolates belonging to nine species of Ophiostomatales were studied: *G. clavigera* (*Gc*), *L. longiclavatum* (*Llo*), *Leptographium terebrantis* (*Lt*), *Grosmannia aurea* (*Ga*), Leptographium wingfieldii (*Lw*), *Grosmannia robusta* (*Gr*), *Grosmannia huntii* (*Gh*), *Leptographium lundbergii* (*Llun*), and *Ophiostoma montium* (Table 1). The identities of many isolates were previously characterized and confirmed using the DNA sequences of rRNA genes and additional protein coding genes (Lim *et al.* 2004). They were cultured and maintained in malt extract agar (MEA) (Tsui *et al.* 2012). Twenty isolates have *MAT1-1* idiomorphs, and 14 isolates have *MAT1-2* idiomorphs.

**Table 1:** Taxa and isolates used to characterize the mating-type (*MAT*) loci

Species	Isolate Code	Geographic Origin	Substrate	Year of Isolation	Idiomorph	GenBank Accession No.
*Grosmannia clavigera* (Gc)	SL-KW1407/UAMH11150 (genome isolate)	Kamloops, Canada	*Pinus contorta*	2001	*MAT1-2*	ACXQ02000000 (locus GL629756)
	B13	Banff, Canada	*Pinus contorta*	2003	*MAT1-2*	JX402933
	SS274	Fairview, Canada	*Pinus contorta × banksiana hybrid*	2007	*MAT1-2*	JX402934
	B101	Banff, Canada	*Pinus contorta*	2003	*MAT1-1*	JX402947
	ATCC18086 (holotype)	Cache Creek, Canada	*Pinus ponderosa*	1965	*MAT1-1*	JX402948
	SS278	Canmore, Canada	*Pinus contorta*	2007	*MAT1-1*	JX402945
	M6	Manning Park, Canada	*Pinus contorta*	2003	*MAT1-1*	JX402943
	M11	Manning Park, Canada	*Pinus contorta*	2003	*MAT1-1*	JX402944
	BW28	Banff, Canada	*Pinus contorta*	2003	*MAT1-1*	JX402946
*Leptographium longiclavatum* (Llo)	SS86	Kakwa, Canada	*Pinus contora*	2007	*MAT1-2*	JX402931
	HV7	Hidden Valley, USA	*Dendroctonus ponderosae*	2003	*MAT1-2*	JX402932
	SS88	Kakwa, Canada	*Pinus contora*	2007	*MAT1-1*	JX402953
	SL-KW1436 (holotype)	Williams Lake, Canada	*Pinus contorta*	2004	*MAT1-1*	JX402954
	HV18	Hidden Valley, USA	*Dendroctonus ponderosae*	2003	*MAT1-1*	JX402955
*Leptographium terebrantis* (Lt)	T26 (LPKRLT-3)	BC, Canada	*Pinus contorta*	2003	*MAT1-2*	JX402936
	T27 (CBS337.7)	Louisiana, USA	*Pinus taeda*	1966	*MAT1-2*	JX402937
	SS394	Fox Creek, Canada	*Pinus contorta × banksiana* hybrid	2007	*MAT1-2*	JX402935
	SS403	Crowsnest Pass, Canada	*Pinus contorta*	2007	*MAT1-1*	JX402956
*Grosmannia aurea* (Ga)	SS419	Grande Prairie, Canada	*Pinus contorta × banksiana* hybrid	2007	*MAT1-2*	JX402938
	SS471	Fox Creek, Canada	*Pinus contorta × banksiana* hybrid	2007	*MAT1-2*	JX402939
	CBS438.69 (OA18-A27) (holotype)	Invermore, Canada	*Pinus contorta var. latifolia*	1969	*MAT1-1*	JX402951
	AU98-Pr2-169	Princeton, Canada	*Pinus contorta*	NA	*MAT1-1*	JX402952
*Leptographium wingfieldii* (Lw)	CMW2095	NA	*Pinus strobus*	2004	*MAT1-1*	JX402950
	CMW2096	NA	*Pinus sylvestris*	NA	*MAT1-1*	JX402949
*Leptographium lundbergii* (Llun)	UAMH9584	Skutskar, Uppland, Sweden	*Pinus sylvestris*	NA	*MAT1-2*	JX402940
	UM1434	NA	NA	2004	*MAT1-1*	JX402941
	DAOM64706	Ontario, Canada	*P. strobus*	1961	*MAT1-1*	JX402958
*Grosmannia huntii* (Gh)	CBS398.77	NY	*P. monticola*	1963	*MAT1-1*	JX402942
	CMW185	South Africa	NA	2001	*MAT1-2*	JX402930
*Grosmannia robusta* (Gr)	CMW668	South Africa	*Picea abies*	2001	*MAT1-1*	JX402957
*Ophiostoma montium* (Om)	UAMH 1363	British Columbia, Canada	*Pinus contorta*	1959	*MAT1-1*	JX402993
	UAMH 4875	Alberta, Canada	*Pinus contorta*	1983	*MAT1-1*	JX402995
	UAMH 11095	Fox Creek, Canada	*Pinus contorta x banksiana* hybrid	2007	*MAT1-2*	JX402994
	UAMH 4838	Alberta, Canada	*Pinus contorta*	1986	*MAT1-2*	JX402996

### DNA extraction, polymerase chain reaction (PCR) amplification, sequencing

**Figure 1:**
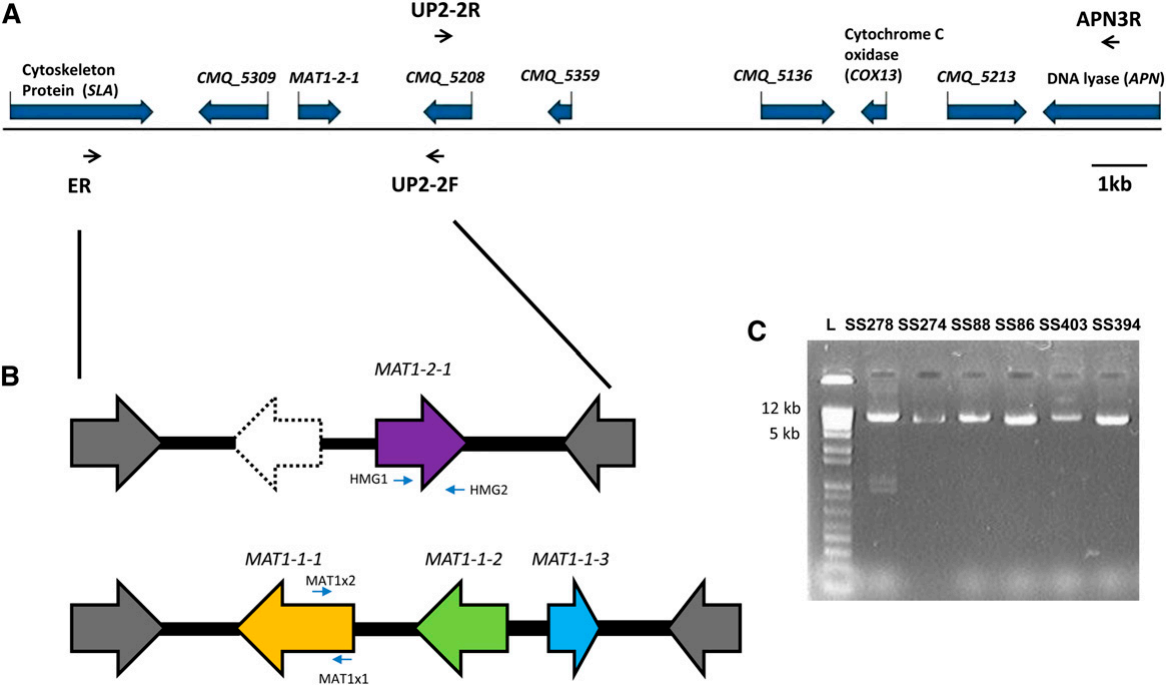
(A) Sequence arrangement and annotation of the *MAT* idiomorph and adjacent genes from annotated genome of *Grosmannia clavigera* SL-KW1407. (B) The *MAT* genes of opposite mating-type isolates as determined from long-range PCR and primer walking using primers ER and UP2-2F. Genes are indicated by different colors, and the arrows indicate the predicted directions of gene translation. (C) Amplicons of *MAT* fragments after long-range PCR were run in an agarose gel to indicate the size variation between *MAT1-2* and *MAT1-1* isolates.

DNA was extracted from all isolates using the procedures described in Roe *et al.* (2011) and Tsui *et al.* (2012). The genome sequencing, assembly, and annotation of *G. clavigera* has been previously described in DiGuistini *et al.* (2011). Based on the genome sequence of *G. clavigera* (GenBank accession number: ACXQ02000000), genes homologous to cytoskeleton assembly protein (*SLA*), HMG-domain of *MAT*, and DNA lyase (*APN*), as well as a few hypothetical proteins without known functions, were identified and characterized (Figure 1) (DiGuistini *et al.* 2011). To characterize the *MAT1-1* idiomorph, we used a long-range PCR amplification approach coupled with primer walking sequencing. Primers ER and UP2-2F targeting the *SLA*, and a hypothetical protein-encoding gene (*CMQ_5208*) located downstream from the *MAT* locus were designed for long-range PCR reaction (Figure 1A) (Table 2). Primers UP2-2R and APN3R, which targeting *CMQ_5208* and *APN*, were also designed to amplify a 15 kb fragment of a putative *MAT1-1* isolate (Figure 1A).

**Table 2:** Major primers used in this investigation

Target Gene	Primer Name	Sequence (5′-3′)
Regular PCR		
* SLA* of *G. clavigera*	ER	GCCACGTCGTTCAACAACTA
Hypothetical protein (CMQ_5309 of	UP2-2F	AGATGGTCATCTCCCGTGAC
* G. clavigera*)		
	UP2-2R	AGATGGTCATCTCCCGTGAC
HMG domain in *G. clavigera*	HMG1	CCGCGCCCACCCAATGCGTACAT
	HMG2	CGAGGGTTGTATCTGTAGTCAGG
alpha-box domain in *G. clavigera*	MAT1x1	CGTCCACTGAATGCCTTCATG
	MAT1x2	GTGGGCAATCATAGCCAAAGT
Cytochrome C oxidase of *G. clavigera*	COX13A	GCTTGACGCAACTATCTCTGC
	COX13B	TGCATCCCCTACTCGATACAC
DNA lyase (*APN*) of *G. clavigera*	cAPNR	GATTCCTTTTACAGCTTTCCCCAC
	APN2R	GACGAGGAGCTGCATCAGG
	APN3F	GACAGGATCACGAACACAACC
	APN3R	TCTTCGATTGGCTCTTTAGGG
* SLA* of *O. montium*	OM7R	CAACACGCTCATTGAGAC
HMG domain in *O. montium*	OM-HMG1	CGCCCCCCCAATGCCTACATTC
	OM-HMG2	CGGGGATTGTACTTGTAGTGCGG
alpha-box domain in *O. montium*	OM-A1	GAATGCCTTCATGGCCTTCC
	OM-A2	ACCTTTGCCATCAACGTCCATTT
Real-time PCR		
truncated *MAT1-1-1*	pMF2	GATCAGATGGGCAAGCTCAG
	pMR2	AAGGCTTGGAAGGACGTGTT

PCR, polymerase chain reaction.

Long-range PCR amplifications of DNA were carried out in 50 μL using a PTC-100 thermocycler (MJ Research Inc., Watertown, MA). Reaction mixtures contained 100 ng of DNA, 1× PCR buffer, 200 μM each dNTP, 0.6 μM of each primer (Eurofins MWG Operon, Huntsville, AL), 1.5 μL of dimethyl sulfoxide and 2 U of Phusion DNA polymerase (Finnzymes; New England BioLabs, Ipswich, MA). The PCR amplifications were performed for 30 sec at 98°, followed by 35 cycles of 10 sec at 98°, 30 sec at 60−62° and 4 min at 72°, and final extension at 72° for 10 min. Sequencing reactions with primer walking were performed at the Centre de recherche du CHUQ, Québec, Canada. Primers for sequencing are listed in (Supporting Information, Table S1).

After *MAT* idiomorph characterization, mating-type specific PCR assay was performed by designing primers HMG1 and HMG2 targeting the HMG domain, as well as primers MAT1x1 and MAT1x2 targeting the α-box domain (Figure 1B, Table 2). Fragments of cytochrome c oxidase subunit gene *VIa* (*COX13*) and *APN* also were amplified for selected species and representatives (Table 2). PCR amplifications were carried out in 25 μL using a PTC-100 thermocycler (MJ Research Inc.). Reaction mixtures contained 20−40 ng DNA, 1× PCR buffer, 200 μM each dNTP, 1.5 μM of each primer (Eurofins MWG Operon), and 1 U of Paq polymerase (Stratagene, Integrated Sciences). The PCR amplifications were performed for 3 min at 94°, followed by 30 cycles of 35 sec at 94°, 35 sec at 52−55°, and 35 sec at 72°, and final extension at 72° for 7 min.

### Gene annotation and analyses

The genome and transcriptomes of *G. clavigera* were assembled and annotated as described in (DiGuistini *et al.* 2011). Gene models were predicted using GLEAN, and the putative gene function assignments were generated from searches of the NCBInr and Swiss-Prot databases using BLAST with PFAM domain assignments (DiGuistini *et al.* 2011).

Sequence reads after primer walking were assembled using the Staden package (Staden 1996) and Geneious Pro (http://www.geneious.com/), and they were compared with genes present in GenBank using BLASTx and BLASTn. The assembled sequences were submitted to FGENESH+ within Softberry (http://www.softberry.ru/berry.phtml?topic=fgenes_plus&group=programs&subgroup=gfs) for gene prediction and to determine the location of the coding/non coding regions, and manually annotated with Artemis (Rutherford *et al.* 2000).

The nucleotide sequences of both *MAT* idiomorphs were compared using dotplot (matrix) analyses implemented in Geneious. The sequences of the *MAT* loci were also compared across different species using the online Artemis Comparison Tool (http://www.webact.org/WebACT/home) with BLASTn algorithm.

### Evolutionary analyses of *MAT* genes

The amino acid sequences encoded by the *MAT1-1* α-box and the *MAT1-2* HMG domain were aligned to sequences of other ascomycetes from GenBank with Clustal W (Thompson *et al.* 1997) implemented in Geneious. Phylogenetic analysis was carried out with the Neighbor-joining (NJ) and maximum likelihood algorithm in MEGA 5 (Tamura *et al.* 2011), PhyML(Dereeper *et al.* 2008) implemented in (http://www.phylogeny.fr/version2_cgi/index.cgi), as well as PRODIST and PROML in PHYLIP 3.69 (Felsenstein 2005).

The nucleotide sequences were aligned in Geneious and manually adjusted with Se-Al v2 (Rambaut 1999) and analyzed with PAUP v4.b10 (Swofford 2003) and MEGA 5 (Tamura *et al.* 2011). Nucleotide data of different genes were subjected to parsimony analysis implemented in PAUP. Bootstrap support for the branches was based on 1000 replicates with TBR branch swapping algorithms and simple sequence addition. The individual data sets were also subjected to NJ implemented in PAUP using GTR model corrections with the proportion of variable sites and gamma shape estimated from Modeltest 3.7 (Posada 2006). Bootstrap values were estimated based on 1000 replicates. Sequences were deposited in GenBank (accession numbers of *MAT* idiomorphs: JX402930-JX402958, JX402993-JX402996; of other genes: JX402959-JX402992).

Signatures of purifying or positive selection acting on the *MAT* genes were tested at the codon level. A maximum likelihood analysis was used to fit codon substitution models to the data using the CODEML program within PAML (Yang 2007). Four random site models were used to describe the variation of ω (= *dN/dS*) among codon sites in an alignment containing the full length *MAT1-1-1* and truncated *MAT1-1-1* genes of six species. Random site models M1A (neutral), M2A (selection), M7 (beta), and M8 (beta and selection) were used to describe the variation of ω among codon sites within each *MAT1* gene. M1a assumes two site classes in proportions p_0_ and p_1_ = 1 − p_0_ with 0 < ω_0_< 1(purifying selection) and ω_1_ = 1 (neutral). M2a adds an additional class of site with ω_2_ as a free parameter, allowing for sites with ω_2_ > 1 (positive selection) with proportion p_2_. M7 is a flexible null model in which a v ratio for each codon is randomly selected from a beta distribution between 0 and 1. M8 adds one additional site class to M7 allowing for positive selection. A test for positive selection was implemented using likelihood ratio tests that compare models pair M1a/M2a (Yang 2007). Three different starting ω values (0.2, 1.0, and 2.0) were implemented for each model fitting, as described in (Joly *et al.* 2010). Codon sites under positive selection were then identified using the empirical Bayes method to calculate the posterior probability that a particular amino acid belongs to a given selection class (neutral, deleterious, or advantageous) (Yang 2007).

### Analysis of mating-type distribution in populations of *G. clavigera* and *L. longiclavatum*

We used the aforementioned mating-type primers (HMG 1, HMG2, MAT1x1, MAT1x2) to identify mating-types in populations from Canada and the USA (Table 2). We performed PCR on DNA extracted from 335 isolates of *G. clavigera* characterized in a previous study (Tsui *et al.* 2012), as well as more than 100 isolates of *L. longiclavatum* currently being investigated (Farfan *et al.* 2011). Mating-type distributions were tested for deviation from the expected ratios of 1:1 using chi-square goodness-of-fit tests.

### Reverse transcriptase (RT) quantitative PCR amplification

RNA was extracted and RT was performed from *G. clavigera* isolate SL-KW1407 cultured on 1% MEA overlaid with cellophane for 4 d (DiGuistini *et al.* 2011) to determine the level of expression from hypothetical protein encoding gene *CMQ_5309* (truncated *MAT1-1-1* gene) and *MAT1-2-1*. RT-quantitative PCR was also carried out with SsoFast EvaGreen Supermix (Bio-Rad) in a volume of 20 µL in a Bio-Rad CFX384 system. Primer pairs were internal to regions of *MAT1-2-1* and truncated *MAT1-1-1* to yield amplicons of 100−120 bp (Table 2). PCRs were described as follows: 96° for 45 sec, followed by 35 cycles of 95° for 15 sec, and 57.5° for 30 s, followed by a melt-curve analysis. The Cq value for amplification of the β-tubulin gene was used as a reference.

## Results

### Organization of the mating-type locus in *G. clavigera*

The *MAT1-2* idiomorph and flanking genes of *G. clavigera* isolate SL-KW1407 were identified from the genome (Figure 1A), and the amplification of the *MAT1-2* idiomorph from isolate *G. clavigera* SS274 demonstrated greater than 99.9% sequence similarity to the sequence of the reference genome. The gene order and orientation near the *MAT* locus was syntenic with other Sordariomycetes. Two ORFs were predicted in the *MAT1-2* idiomorph. One of the translated proteins bearing the HMG domain (285 amino acids; EFX05114) was homologous to the MAT1-2-1 of other ascomycetes and shared 65% similarity to that of *Ophiostoma himal-ulmi*. In contrast, *CMQ_5309*, encoding a hypothetical protein (EFX04946.1), had no significant similarity to any genes in the NCBI sequence database. *SLA2*, which encodes the cytoskeleton assembly control protein (EFX04935.1), was located upstream from the *MAT* locus, whereas cytochrome c oxidase subunit (EFX05020.1) gene *COX13* and DNA lyase (EFX04950.1) gene *APN2* were located downstream from the MAT (Figure 1A). The deduced amino acids of *SLA* and *APN* had 68% and 62% identities, respectively, to those of *Neurospora crassa*, a relative in the Sordariomycetes. However, the intergenic distance between *MAT* and *APN* loci in *G. clavigera* was large, spanning greater than 10 kb with several putative proteins coding genes of unknown functions identified (Figure 1A). One of the putative proteins (CMQ_5213; EFX04951) had a protease-associated domain and was homologous (35% similarity) to a RING-9 protein in *Verticillium albo-atrum* (XP_003007854). The functions of these additional genes to mating activity are not yet established. tBLAST searches using the α-box domain containing genes from different ascomycetes as queries did not return any significant matches in the SL-KW1407 genome, confirming the absence of *MAT1-1-1* gene in the reference isolate.

Using the primer walking approach, we obtained the full sequence (22,910 bp) of the *MAT1-1* idiomorph of *G. clavigera* isolate B101. The gene arrangement was syntenic to the assembled genome of *G. clavigera* isolate SL-KW1407. The genes of *SLA*, *MAT*, *COX13*, and *APN* were located in the identical order and orientation in both *MAT1-1* and *MAT1-2* idiomorphs. At the nucleotide level, the sequence of the >15-kb fragment spanning from hypothetical protein coding gene *CMQ_5208* to *APN* was 99.9% identical in isolates B101 and SL-KW1407.

Three open reading frames (ORFs) were predicted in the *MAT1-1* idiomorph of isolate B101, encoding proteins of 619, 340, and 171 amino acids, respectively (Figure 1B). The first protein had α-box domain and was homologous (52% similarity) to the *MAT1-1-1* (ACZ53927.1) of *Ophiostoma novo-ulmi* subsp. *novo-ulmi*. The second and third proteins were also 24% and 52% similar to the *MAT1-1-2* (ACZ53926.1) and *MAT1-1-3* (ACZ53925.1) of *O*. *novo-ulmi* subsp. *novo-ulmi*, respectively.

**Figure 2:**
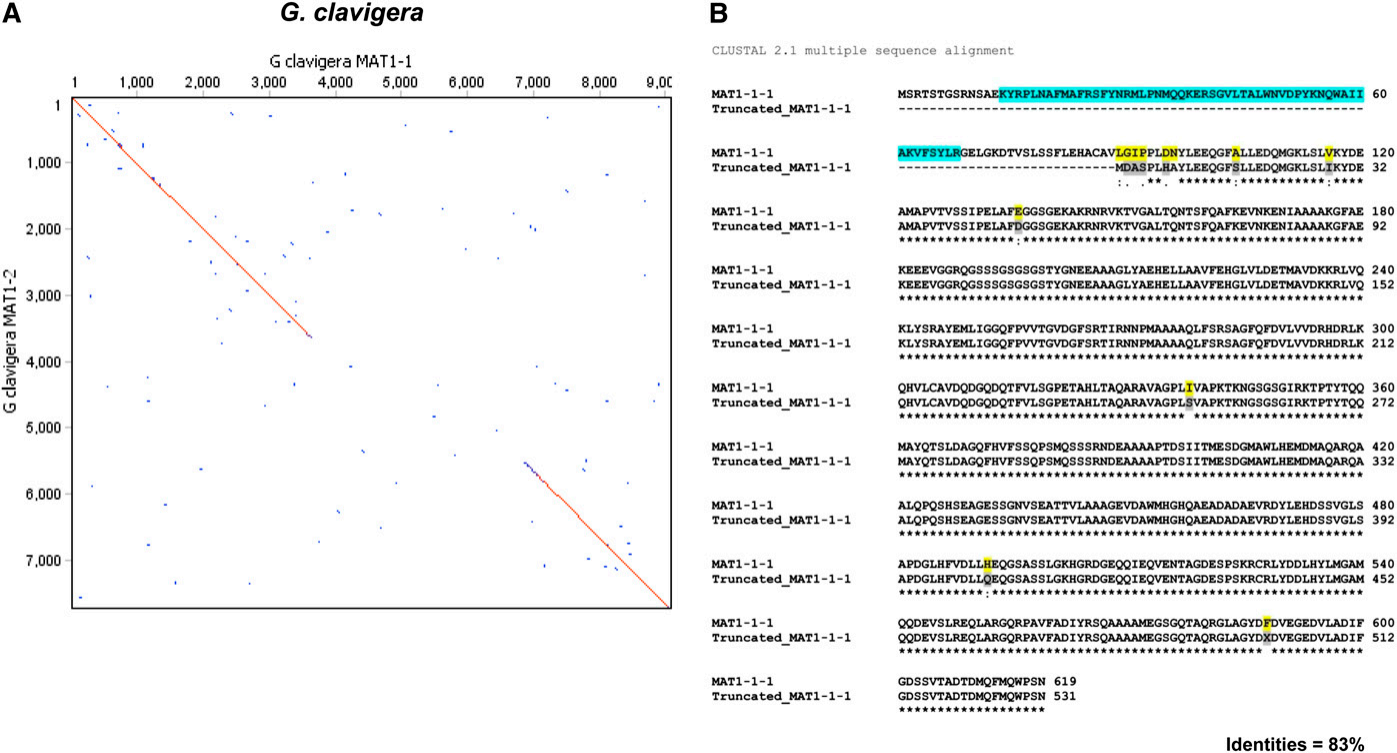
Comparison of *MAT* loci in *Grosmannia clavigera*. (A) Dotplot comparison/pairwise alignment of DNA sequence data for *MAT1-1* and *MAT1-2* idiomorphs of *G. clavigera*. Sequence lengths are given along the axes. (B) The amino acid alignment of MAT1-1-1 in *MAT1-2* idiomorph to the truncated MAT1-1-1 in *MAT1-2* idiomorph of *G. clavigera* by Clustal W. The comparison indicates the deletion of α-box domain (in square) in truncated MAT1-1-1.

Dotplot comparison of the *MAT1-1* and *MAT1-2* idiomorphs revealed nucleotide sequence similarity (99%) in the SLA encoding gene, regions upstream from the *MAT* locus, as well as the gene encoding protein CMQ_5208 (Figure 2A). Surprisingly the gene encoding putative protein (CMQ_5309; EFX05047.1) located upstream from the *MAT1-2-1* gene in isolate SL-KW1407 was homologous (>80% similarity in amino acids) to the *MAT1-1-1* gene in the *MAT1-1* idiomorph but shorter in length and without introns. Sequence comparison revealed α-box domain truncation/deletion (89 amino acids) at the N-terminus of the putative protein. The start codon was, however, present in the putative protein coding sequence (now called truncated MAT1-1-1) followed by five codons that are not homologous to the *MAT1-1-1* on the *MAT1-1* idiomorph (Figure 2B). This 15bp 5′ end sequence was used to search the *G. clavigera* genome as well as the NCBI database but no significant match was returned.

The *MAT* loci of additional *G. clavigera* isolates from different geographic locations were also sequenced using the identical long-range PCR approach to investigate whether the truncated *MAT1-1-1* gene could be unique solely to isolate SL-KW1407 (Table 1). Sequencing data confirmed that the isolates bearing the same mating-type have identical idiomorph size (data not shown). The presence of this truncated gene in multiple *G. clavigera* isolates confirms that it is not a spurious result or a unique feature of the reference isolate.

### Organization of the *MAT* idiomorphs in fungal species related to *G. clavigera*

The presence of the truncated/incomplete gene suggested that the ancestor of *G. clavigera* may have had both *MAT1-1* and *MAT1-2* copies and was homothallic. To understand the evolutionary history leading to the presence of the truncated *MAT1-1-1* gene, we used the same primer pair ER and UP2-2F (targeting *SLA* and hypothetical protein-coding gene *CMQ_5208*) to characterize the *MAT* loci of several fungi related to *G. clavigera* (Lim *et al.* 2004) (Table 1). If the truncated gene has been inherited from a common ancestor, it may be preserved in other related species since it may confer an evolutionary or ecological advantage.

Sequences containing orthologous genes of the *MAT* locus and flanking regions were characterized from 21 isolates belonging to seven species within the Ophiostomatales: *Llo*, *Lw*, *Lt*, *Llun*, *Ga*, *Gr*, and *Gh* (Table 1, Figure 1C). Interspecific variations in fragment size ranged from 5787 to 8011 bp in *MAT1-1* isolates, and from 5209 to 7727 bp in *MAT1-2* isolates among different species. The variations in fragment size are also partly due to incomplete sequencing of the flanking sequences of *SLA* and the putative protein-coding gene *CMQ_5208*. The 3′end of mating-type loci in *Llun* and *Gh* were incomplete because the primers targeting the *CMQ_5208* failed to amplify, and a new primer GCM3 was designed to target the conserved regions in the MAT1-1-3 for *MAT1-1* idiomorph characterization. Conversely, primer HMG2 targeting *MAT1-2-1* gene was used to amplify the partial *MAT1-2* idiomorph fragment. Only the *MAT1-1* idiomorphs of *Lw* and *Gr* were obtained because *MAT1-2* isolates were not available (Table 1). The *MAT1-1* idiomorph of *L. lundbergii* isolate DAOM64706 was shorter and incomplete when compared with other taxa, so its sequence was not included in the analyses.

**Figure 3:**
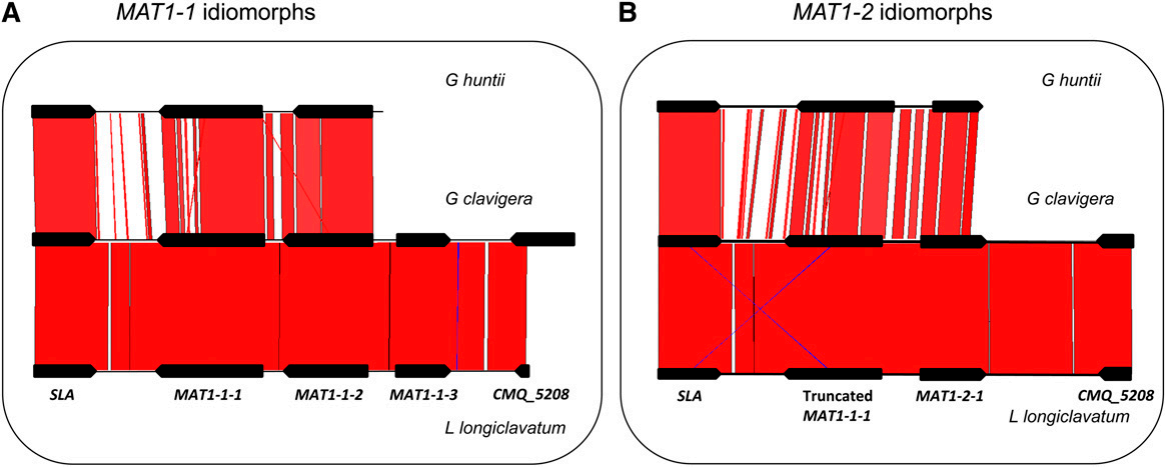
Homology among the *MAT* locus. (A) *MAT1-1* idiomorph; (B) *MAT1-2* idiomorph, of *G. huntii*, *G. clavigera*, and *L. longiclavatum*. The diagram was prepared from the output of Artemis Comparison Tool. Regions of strong homology are shaded and connected by lines. The intensity of shading indicates the strength of homology. Genes are represented by box arrows. The *MAT1-2* sequence of *G. clavigera* SL-KW1407 was obtained from the genome sequence, while the *MAT1-1* sequence of *G. clavigera* and opposite *MAT* isolates of other fungi were obtained in this investigation.

Dotplot comparison of the *MAT1-1* and *MAT1-2* idiomorphs in each of the species also revealed nucleotide sequence similarity (95–99%) in the SLA protein-coding regions, regions upstream from the *MAT* locus, as well as the *CMQ_5208* (Figure S1). The organization of the idiomorphs among these seven species was identical, with the presence of three predicted ORFs in the *MAT1-1* idiomorph and two ORFs in the *MAT1-2* idiomorph, in addition to the truncated *MAT1-1-1* gene (the comparison among *Gh*, *Gc*, and *Llo* is illustrated in Figure 3). The nucleotide sequence of the *MAT* idiomorphs was similar among these seven species within *Grosmannia*: it ranged from 72% between *G. clavigera* and *G. huntii* to 97.5% between *G. clavigera* and *L. longiclavatum* (Table S2 and Figure 3, A and B). Sequence similarity was much lower when compared to other ascomycetes.

**Figure 4:**
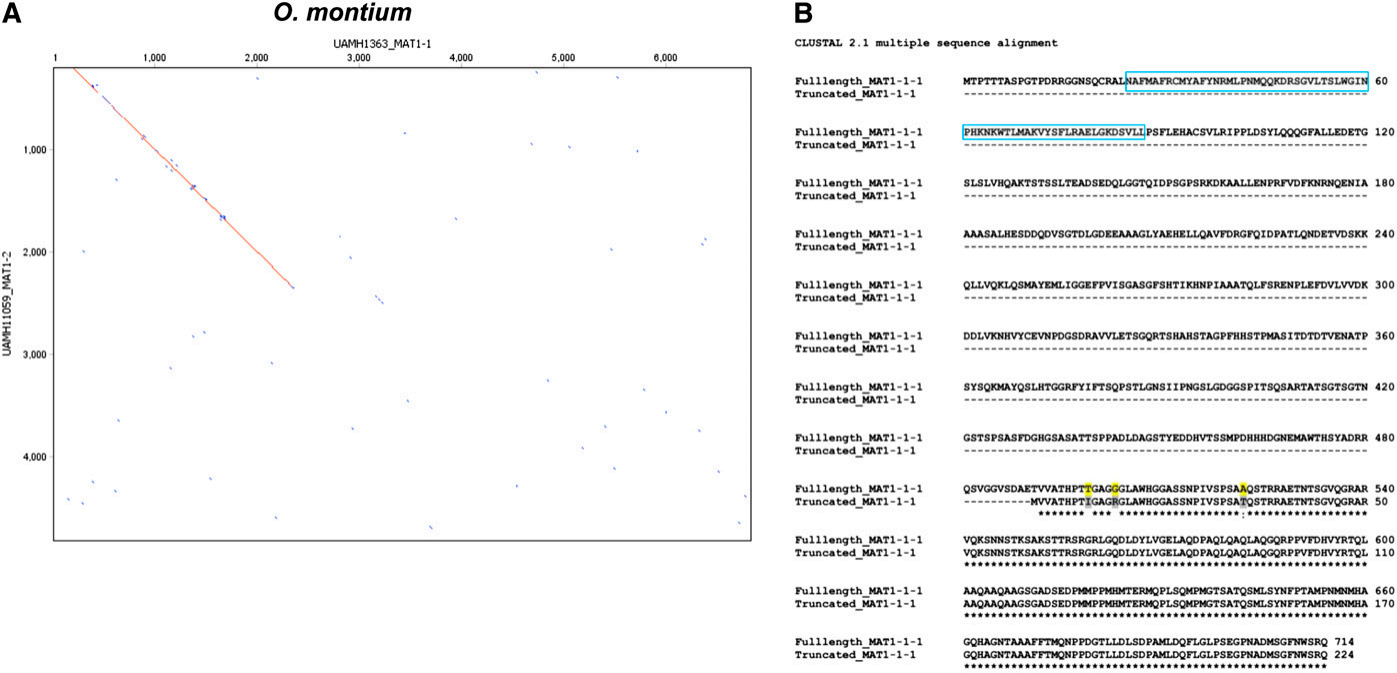
Comparison of *MAT* loci in *Ophiostoma montium*. (A) Dotplot comparison/pairwise alignment of DNA sequence data for *MAT1-1* and *MAT1-2* idiomorphs of *O. montium*. Sequence lengths are given along the axes. (B) The amino acid alignment of MAT1-1-1 in *MAT1-2* idiomorph to the truncated MAT1-1-1 in *MAT1-2* idiomorph of *O. montium* by Clustal W. The comparison indicates the truncated MAT1-1-1 was highly eroded and the absence of α-box domain (in square in full length MAT1-1-1).

*Ophiostoma* is a sister genus to *Grosmannia* (Zipfel *et al.* 2006). A genomic DNA library of *O. montium* populations (R. C. Hamelin, unpublished data) was searched to design primers anchoring the *SLA* and the flanking regions of the *MAT* locus in both mating-types. Using a long-range PCR amplification and primer walking approach, we also amplified the *MAT* locus from four isolates of *O. montium* including both mating-types (Tables 1 and 2). We found fragments of ca. 6 kb and 5 kb in size that corresponded to both *MAT1-1* and *MAT1-2* idiomorphs. Dotplot comparison of the *MAT1-1* and *MAT1-2* idiomorphs were similar to the orthologs from *Grosmannia* species and revealed nucleotide sequence similarity in the SLA protein and intergenic regions upstream from the *MAT* locus (Figure 4A). The *MAT1-1* idiomorph had three putative ORFs that corresponded to *MAT1-1-1*, *MAT1-1-2*, and *MAT1-1-3* genes, and they were similar (53–76% at amino acids level) to those of *O. novo-ulmi* subsp. *novo-ulmi* (Figure S2). The *MAT1-2* idiomorph contained a *MAT1-2-1* gene encoding a protein (263 aa) with HMG domain (75% similar to that of *O. novo-ulmi* subsp. *novo-ulmi)*, as well as a truncated *MAT1-1-1* gene without the α-box domain located upstream from the *MAT1-2-1* gene. The truncated MAT1-1-1 (224 amino acids) was short when compared with the original MAT1-1-1 (714 aa) in the *MAT1-2* idiomorph (Figure 4B) and the extent of deletion/truncation was greater than that in *Grosmannia* species.

### Evolutionary analyses of the *MAT* loci

**Figure 5:**
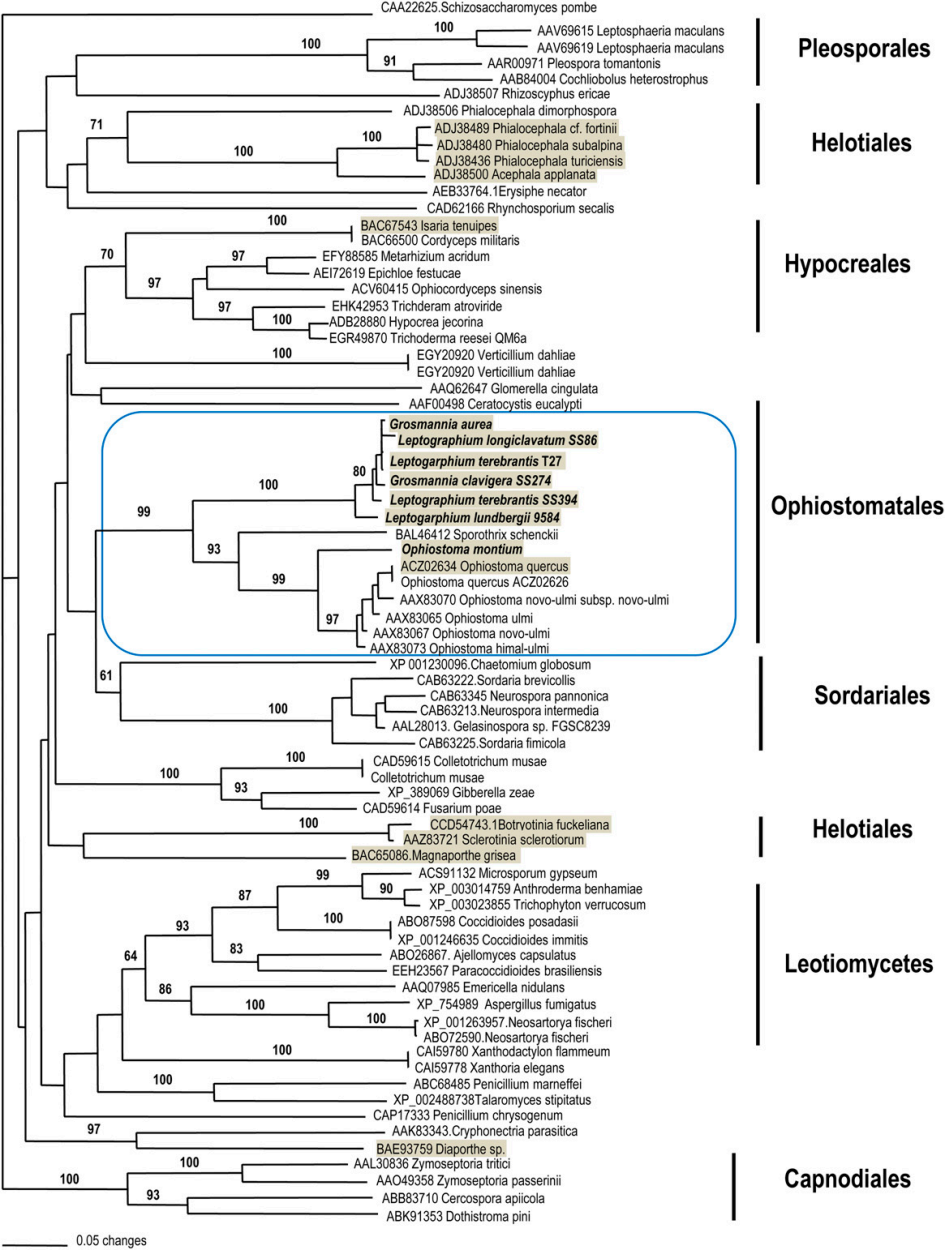
NJ tree generated from MEGA showing the phylogenetic relationships among ascomycetes inferred from the HMG domain (129 amino acid characters) of the MAT1-2-1. Number on branches indicated bootstrap support (1000 pseudoreplicates) more than 60% from NJ and PhyML (from left to right). The taxa shaded in gray indicate the presence of truncated *MAT* genes in opposite *MAT* isolates.

Phylogenetic analysis inferred from 129 amino acid characters from HMG-domain of 74 ascomycetes supported the monophyletic origin of fungi in Ophiostomatales, and *Grosmannia* and *Ophiostoma* formed a sister relationship with strong statistical support (Figure 5). Representatives of these two genera also clustered with the members of *Neurospora* and *Sordaria* in Sordariomycetes, with strong likelihood support. Within the cluster of Ophiostomatales, *G. clavigera*, *L. longiclavatum*, and *L. terebrantis* together with either *G. aurea* or *L. wingfieldii* formed a monophyletic clade with >90% bootstrap support. *O. montium* formed a sister relationship to a cluster containing *O. novo-ulmi* and its relatives. *Sporothrix schenckii*, a human pathogen, also nested within the same cluster with strong support (93%). Sequence analysis of α-box domain from 39 ascomycetes species also supported the same sister relationship between *Grosmannia* and *Ophiostoma* but the relationships among the major ascomycete families were not well resolved (Figure S3).

*MAT* genes have been useful for evaluating the phylogenetic relationships among different fungal species (Debuchy and Turgeon 2006; Devier *et al.* 2009). Gene genealogies of *MAT1-1-2*, *MAT1-1-3*, and *MAT1-2-1* (Figure S4) were concordant with previous species relationships established based on rRNA and other protein coding genes (Lim *et al.* 2004). Also, phylogenetic analyses of *SLA*, intergenic regions upstream of full-length *MAT1-1-1* and truncated *MAT1-1-1*, *COX13*, and *APN*, demonstrated that sequences of opposite mating-types corroborate species phylogenies rather than showing *trans*-specific polymorphism (Figure S5). There were no strong conflicts in tree topologies inferred from genes at and flanking the MAT locus. Also there was no conflict in topologies among various tree-building algorithms.

**Figure 6:**
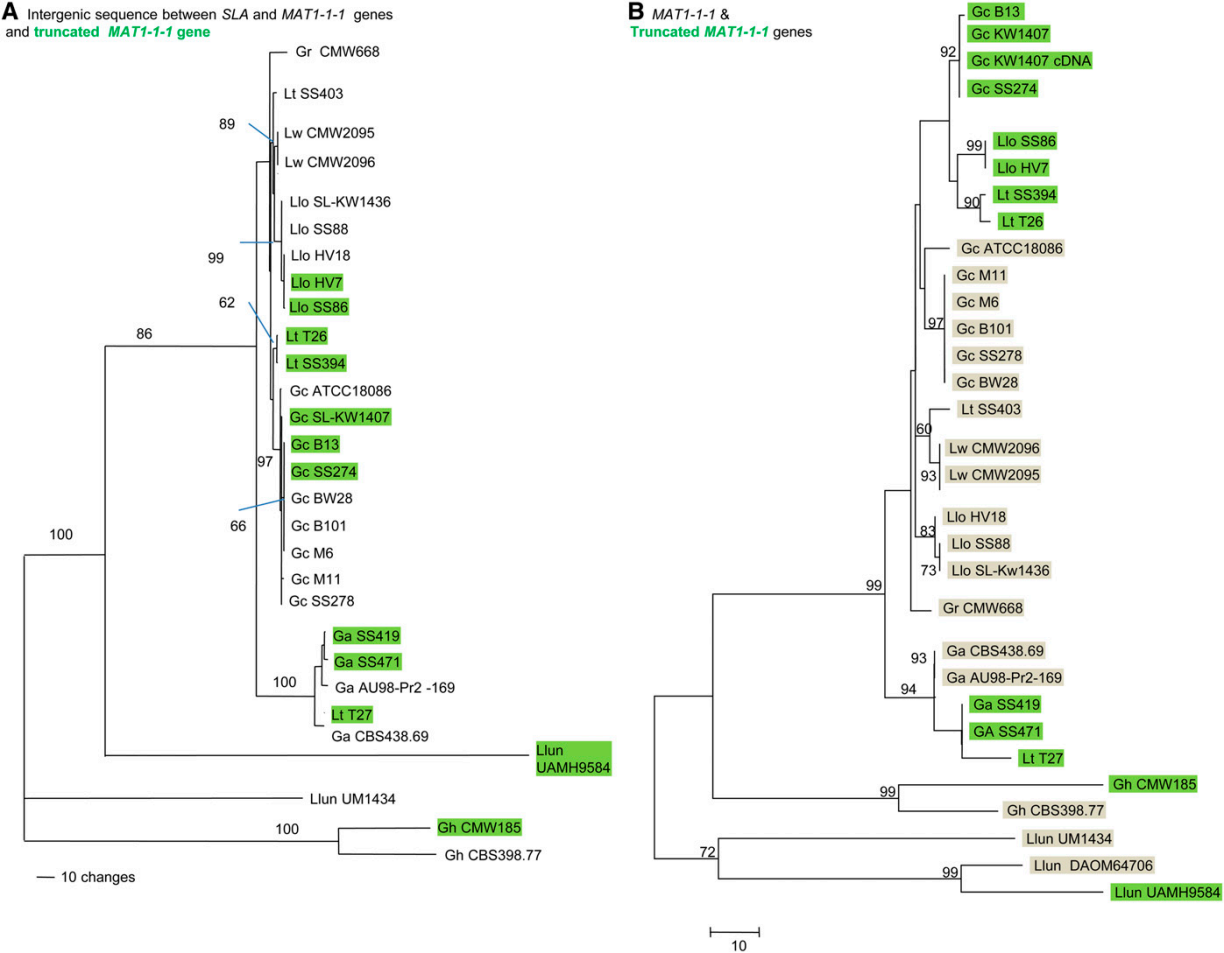
Gene genealogies showing the phylogenetic relationships between *G. clavigera* and relatives. (A) Intergenic sequences after 3′ end of *SLA* to the 5′ end of the truncated *MAT1-1-1* and *MAT1-1-1* genes (1349 characters), and (B) the full-length and truncated *MAT1-1-1* genes. *MAT1-2* isolates are indicated in green while *MAT1-1* isolates are highlighted in gray (1968 characters). Number on branches indicated bootstrap support (500 pseudoreplicates) greater than 60%.

Phylogenetic analyses of the nucleotide sequences inferred that recombination occurred in the flanking sequences upstream from *MAT1-1-1* and truncated *MAT1-1-1* genes (Figure 6, A and B). The tree topology of flanking sequence did not conflict with that of SLA coding sequences (Figure S5) because representatives of both mating-types clustered within a species (Figure 6A). Without recombination, the flanking sequence on *MAT1-1* idiomorph would be expected to cluster separately to the flanking sequence on the *MAT1-2* idiomorph in the gene genealogy. The nucleotide sequence of truncated *MAT1-1-1* genes diverged from the full-length *MAT1-1-1* genes as a result of independent accumulation of mutations (Figure 6B). Sequences of the opposite mating-type within a species did not cluster together as for the *SLA* gene, but were divergent even though they had high similarity (83%) at the amino acid level.

Based on the tests of selection using likelihood ratio tests, diversifying (positive) selection was detected in the truncated *MAT1-1-1* from the full-length *MAT1-1-1* at the intraspecific level (Tables 3, A and B). The truncated *MAT1-1-1* genes in the *MAT1-2* idiomorph are under positive selection from the full-length *MAT1-1-1* genes on the *MAT1-1* idiomorph in *G. clavigera*, *L. longiclavatum* and *G. aurea*, but not *L. terebrantis* (Tables 3, A and B). The truncated/incomplete *MAT1-1-1* gene may go through neutral evolution due to loss of function or adaptive evolution. In contrast, tests of selection on *MAT* genes (*MAT1-2-1*, *MAT1-1-1*, and truncated *MAT1-1-1* individually) at the interspecific level revealed purifying selection (Table S3C), indicating that these genes are preserved for proper function of the sexual cycle.

**Table 3:** Parameter estimates and likelihood values of the various models of codon evolution using CODEML in PAML

	Model	Model Parameters	-lnL	Models Comparison	2ΔL	*Pr.*	Sites Under Positive Selection (Bayes Empirical Bayes, Pr. ω>1)
**A. Test on the truncated and full-length *MAT1-1-1* genes within a species (datasets with positive selection detected)**							
*G. clavigera* (9 isolates)	M1a (neutral)	*P_0_* = 0.26, *P_1_* = 0,74	2172.75	M1a *vs.* M2a	17.70	<0.001	2 G (0.99**), 3 I (0.99**), 8 N (0.99**)
	M2a (selection)	*P_0_* = 0.99, *P_1_* = 0.00, *P_2_* = 0.004, ω_2_ = 227.05	2163.90				
	M7 (beta)	*P* = 2.30, *q* = 0.005	2172.75	M7 *vs.* M8	17.41	<0.001	2 G (0.99**), 3 I (0.99**), 8 N (0.99**)
	M8 (beta + ω)	*P_0_* = 0.98, *P* = 0.005, q = 3.27, ***P_1_* = 0.02, ω = 81.42**	2164.05				
*G. aurea* (four isolates)	M1a (neutral)	*P_0_* = 0.77, *P_1_* = 0.23	2102.86	M1a *vs.* M2a	21.18	<0.001	3 A (0.94), 8 A (0.94)
	M2a (selection)	*P_0_* = 0.99, *P_1_* = 0.00, *P_2_* = 0.003, ω_2_ = 282.78	2092.27				
	M7 (beta)	*P* = 0.005, *q* = 0,012	2102.96	M7 *vs.* M8	21.37	<0.001	3 A (0.97*), 8 A (0.97*)
	M8 (beta + ω)	*P_0_* = 0.99, *P* = 0.005, q = 15.47, ***P_1_* = 0.003, ω = 282.82**	2092.27				
*L. longiclavatum* (five isolates)	M1a (neutral)	*P_0_* = 0.72, *P_1_* = 0.28	2183.24	M1a *vs.* M2a	18.88	<0.001	3 A (0.93), 8 A (0,93)
	M2a (selection)	*P_0_* = 0.99, *P_1_* = 0.00, *P_2_* = 0.002, ω_2_= 209.51	2173.80				
	M7 (beta)	*P* = 0.005, *q*= 0.011	2183.25	M7 *vs.* M8	18.90	<0.001	3 A (0.97*), 8 A (0.97*)
	M8 (beta + ω)	*P_0_* = 0.99, *P* = 0.51, q = 1.49, ***P_1_* = 0.001, ω = 209.58**	2173.80				

**B. Test on the truncated and full-length *MAT1-1-1* genes within a species (dataset without positive selection detected)**							
*L. terebrantis* (4 isolates)	M1a (neutral)		2462.37	M1a *vs.* M2a	1.30	ns	
	M2a (selection)		2461.72				
	M7 (beta)		2462.54	M7 *vs.* M8	1.65	ns	
	M8 (beta + ω)		2461.72				

Parameter estimates and likelihood values of the various models of codon evolution using CODEML in PAML. Notes *for G. clavigera*: Models assuming positive selection (M2a and M8) fit better the data than neutral models (M1a and M7) according to likelihood ratio tests. Model M2 assumes that 0.4% of the sites have *dN/dS* value = 227.05. Three sites under strong positive selection (*P* > 0.99) are identified by this model with Bayes empirical Bayes methods. Model M8 showed that approximately 98% of sites have *dN/dS* from a U-shaped beta distribution (hence, data fit strongly this model) and approximately 2% of site are under strong positive selection with *dN/dS* = 81.4. Both models M2 and M8 identified the same positive selection sites with Bayes empirical Bayes methods (even if model M8 would assume more sites under positive selection).

### Transcript analysis of *MAT* genes

The *MAT1-1-1* gene with α-box domain is a master regulator of sexual reproduction and is involved in gamete fertilization and the formation of ascogenous hyphae; the deletion of *MAT1-1-1* gene can lead to incomplete development of perithecia in some ascomycetes (Debuchy *et al.* 2010). To determine whether the truncated *MAT1-1-1* gene is transcribed, PCR and RT-qPCR experiments were performed on the cDNA. The *MAT1-2-1* gene (Cq = 28) and truncated *MAT1-1-1* gene (Cq = 32.9) were expressed during the vegetative stage in mycelia (4d culture with cellophane overlaid on MEA plates), even though the expression level was low compared with the reference β-tubulin gene (Cq = 25.5). The data were also consistent with the low level of sex gene expression reported in transcriptomic data in various terpenoid compound treatments (DiGuistini *et al.* 2011) (Table S4).

### Determination of MAT type ratio in populations of *G. clavigera* and *L. longiclavatum*

Using specific primers targeting the α-domain and HMG domain, we detected both the *MAT1-1* and *MAT1-2* idiomorphs in *G. clavigera*. We tested the null hypothesis of balanced numbers of the two *MAT* idiomorphs within the epidemic population samples in Tsui *et al.* (2012). Both mating-types were present in the populations at all of the spatial scales. *MAT1-1* and *MAT1-2* frequencies did not significantly deviate from a 1:1 ratio in any of the 19 populations, the four genetic clusters inferred from Bayesian estimation during population genetic studies, nor the entire population (*P* < 0.05) (Table 4A). Similarly both *MAT1-1* and *MAT1-2* also were detected in populations of *L. longiclavatum*, and its mating-type ratio did not deviate significantly from 1:1 at small spatial scale (Table 4B). However the MAT ratio was significantly different at larger landscape level, where *MAT1-1* isolates appeared more frequent in the Rocky Mountain (Cluster Rocky) whereas *MAT1-2* isolates were predominant in the new epidemics area (Cluster North; northern British Columbia and Alberta) (Table 4B).

**Table 4:** MAT ratio tests on populations of *Grosmannia clavigera* and *Leptographium longiclavatum*

Population (Location, Province, or State)	Number Total	Clone Corrected (293 Isolates)		
		*MAT1-1*	*MAT1-2*	χ2 (*P* Value)
A. MAT ratio of *Grosmannia clavigera* population, including location and sample size (after clone correction) (*p< 0.05)				
Houston, BC	16	5	11	2.25, *P* = 0.133
Fort St. James, BC	23	13	10	0.39, *P* = 0.532
Tumbler Ridge, BC	13	6	7	0.0769, *P* = 0.7815
Fairview, BC	11	4	7	0.8182, *P* = 0.3657
Grande Prairie, AB	20	8	12	0.8, 0.3722
Fox Creek, AB	12	5	7	0.333, 0.5637
Kakwa, AB	17	5	12	2.8824, 0.08956
Valemount, BC	8	4	4	0,1
Williams Lake, BC	15	4	11	3.2667, 0.0707
Manning Park, BC	19	10	9	0.0526, 0.8185
Golden, BC	8	4	4	0,1
Yoho, BC	7	3	4	0.1429, 0.7055
Banff, AB	20	11	9	0.2, 0.6547
Canmore, AB	39	14	25	3.1026, 0.07817
Cypress Hills, AB	5	2	3	0.2, 0.6547
Sparwood, BC	7	3	4	0.1429,0.7055
Crowsnest Pass, AB	9	5	4	0.1111,0.7389
Hidden Valley, MT, USA	20	11	9	0.2, 0.6547
Hell Roaring, ID, USA	24	15	9	1.5, 0.2207
**Total**	293	132	161	2.8703, 0.09023
Genetic clusters inferred from Tsui *et al.* (2012)				
NBC	39	18	21	0.231,0.63
NORTH	81	32	49	3.57, 0.059
SBC	34	14	20	1.059, 0.303
ROCKY	139	68	71	0.065, 0799

B. MAT ratio of *Leptogaphium longiclavatum* populations, including locations and sample size (after clone correction) (*p<0.05)				
Canmore	15	4	11	3.27, 0.07
Crownsnest Pass	6	2	4	0.667, 0.414
Cypress Hills	2	1	1	0, 1
Golden	8	3	5	0.5, 0.48
Sparwood	7	3	4	0.143, 0.705
Yoho	5	2	3	0.2, 0.655
Cluster Rocky	43	15	28	3.93, 0.047*
Fairview	15	9	6	0.6, 0.439
Fox Creek	21	12	9	0.429, 0.512
Grande Prairies	29	19	10	2.793, 0.09
Kakwa	22	11	11	0, 1
Tumbler Ridge	26	16	10	1.385, 0.239
Valemount	7	4	3	0.143, 0.705
Cluster North	120	71	49	4.033, 0.045*
**Total**	163	86	77	0.497, 0.48

MAT, mating type.

## Discussion

### The *MAT* locus organization indicates heterothallism

All fungal species in this study are heterothallic because they have a locus with one of the two alternative single-copy idiomorphs, *MAT1-1* or *MAT1-2*. The organization of opposite *MAT* loci was highly conserved among *L. longiclavatum*, *L. terebrantis*, *G. aurea*, *G. huntii*, *L. lundbergii*, *L. wingfieldii*, and *O. montium*, thus supporting a common origin in Ophiostomatales (Butler 2007). Also the general *SLA-MAT-APN* pattern, as well as the synteny and orientation of *MAT1-1-1*, *MAT1-1-2*, and *MAT1-1-3* genes on *MAT1-1* idiomorph are consistent with other representatives within Sordariomycetes, such as *Neurospora*, and *Podospora* (Debuchy and Turgeon 2006). This suggests a common evolutionary origin of the *MAT* organization/structure for members of the Ophiostomatales and even the Sordariomycetes.

Whether heterothallism is the ancestral state in ascomycetes has been a major biological question in fungal evolution (Coppin *et al.* 1997; Butler 2007). Evolution from heterothallism (outcrossing) to homothallism (haploid selfing) could be the most likely scenario based on population genetics models (Nauta and Hoekstra 1992; Billiard *et al.* 2011). Recent analyses of mating-type evolution have ascertained *Neurospora* to be heterothallic in ancestry (Strandberg *et al.* 2010; Nygren *et al.* 2011; Gioti *et al.* 2012). We speculate that the representative of *Grosmannia* and *Ophiostoma* may have diverged from the same common heterothallic ancestor as *Neurospora*. However, it is important to determine the mating-type organization from additional species in Ophiostomatales because their mating systems and breeding strategies (Wilken *et al.* 2012) may be different from other Sordariomycetes.

Another special feature in the *MAT* locus is that *COX13* is located between *MAT* and *APN* genes in *G. clavigera*, and this is similar to the organization found in the human pathogen *Histoplasma capsulatum* (Fraser *et al.* 2007). Although the *MAT* and *APN* loci were at least 15 kb apart, there was no evidence to suggest the presence of transposable elements, which have been reported in *MAT* locus expansion in the obligate biotroph *Blumeria graminis* (Spanu *et al.* 2010), *H. capsulatum* (Fraser *et al.* 2007) and the endophyte *Phialocephala fortiniii* (Zaffarano *et al.* 2010).

### Unequal recombination defined the evolution of the truncated *MAT1-1-1* genes in *Grosmannia* and *Ophiostoma*

Most fungi in this investigation carried the truncated *MAT1-1-1* in the *MAT1-2* idiomorphs. The polymorphism between full-length and truncated *MAT1-1-1* indicated that the event leading to the current *MAT* loci organization was ancient—before the radiation of *G. clavigera* from other species. A number of competing scenarios may account for the evolutionary origin of this unique organization.

**Figure 7:**
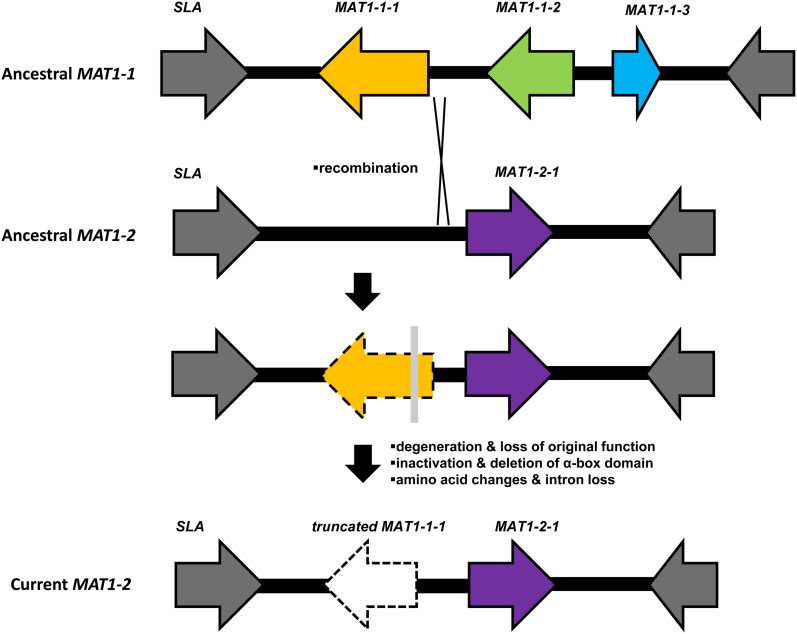
Proposed model for the evolution of *MAT* locus in the common ancestor of *Grosmannia* and *Ophiostoma* (Ophiostomatales).

Unequal recombination/crossover at the *MAT* locus between opposite mating-type idiomorphs during the pairing of chromosomes should be the most favorable mechanism to account for the truncated *MAT1-1-1* (Gioti *et al.* 2012) (Figure 7). The ancestral *MAT1-2* idiomorph of *Grosmannia* and *Ophiostoma* members contained one ORF corresponding to the *MAT1-2-1* gene bearing the HMG domain. A fragment of the *MAT1-1* idiomorph, for instance the *MAT1-1-1* gene and the 5′end flanking sequence, could have become integrated into the ancestral *MAT1-2* idiomorph during the crossover in sexual reproduction (Figure 7). Afterward the α-box domain had been deleted over evolutionary time (Figure 7).

Unequal recombination at the *MAT* locus is not unique to members of *Grosmannia* and *Ophiostoma*. This event has clearly happened many times, and its footprints have been demonstrated in other ascomycete species (Yokoyama *et al.* 2003; Paoletti *et al.* 2005b; Seidl *et al.* 2009; Amselem *et al.* 2011; Wilken *et al.* 2012) (Figure 5). Fragments of the *MAT1-1-1* and *MAT1-1-3* genes were reported in 10 isolates of *O. quercus* that contained the full, complete *MAT1-2-1* gene in their *MAT1-2* idiomorphs (Wilken *et al.* 2012). Similarly, truncated *MAT1-1-1* genes were identified in the *MAT1-2* idiomorphs of at least five *Phialocephala* species (Zaffarano *et al.* 2010) and the one in *Hypocrea jecorina* (Seidl *et al.* 2009). In contrast, a partial *MAT1-2-1* sequence was found in the *MAT1-1* idiomorph of *Aspergillus fumigatus* (Paoletti *et al.* 2005b). Also fragments of homologous *MAT1-2-1* and *MAT1-1-1* genes were detected bordering the mating-type idiomorphs in *Botrytis cinerea* isolates (Amselem *et al.* 2011).

Recombination is supposed to be suppressed and rare at the *MAT* locus in ascomycetes (Idnurm 2011), but recombination or crossover events have been reported. Homologs of *MAT1-1-2* and *MAT1-1-3* also were reported in the *MAT1-2* idiomorph of *Diaporthe* W- and G-type species, possibly due to recombination of idiomorphs (Kanematsu *et al.* 2007). The *MAT1-2-2* gene in the *MAT1-2* idiomorph of *Magnaporthe oryzae* was partially homologous to the *MAT1-1-3* gene in opposite mating-types (Kanamori *et al.* 2007). Recombination breakpoints and unequal crossover events have been revealed in the *MAT* loci of *Neurospora*, *Cochliobolus*, *Stemphylium*, *Ascochyta* and *Phoma* during the comparison of *MAT* idiomorphs between heterothallic to homothallic representatives (Yun *et al.* 1999; Inderbitzin *et al.* 2005; Gioti *et al.* 2012; Woudenberg *et al.* 2012).

Acquisition of *MAT* genes by transposition has been independently reported in several ascomycetes (Rydholm *et al.* 2007; Poggeler *et al.* 2011; Gioti *et al.* 2012). Therefore, the intronless *MAT1-1-1* in *MAT1-2* idiomorph may have arisen by retrotransposition from the *MAT1-1-1* cDNA from the ancestral *MAT1-1* idiomorph, but the *MAT* loci do not contain transposon-related and repetitive sequences. Interspecific introgression of *MAT1-1-1* gene and vegetative incompatibility genes from *Ophiostoma ulmi* into *O. novo-ulmi* has been proposed (Paoletti *et al.* 2006). Introgression or non-random acquisition of *MAT* genes was also demonstrated in the evolutionary histories among multiple *Neurospora* species (Menkis *et al.* 2010; Strandberg *et al.* 2010). However, the molecular data do not support introgression to account for the presence of the truncated *MAT1-1-1* gene because the gene genealogies of *MAT* and flanking genes do not contradict the species phylogeny of *Grosmannia* and *Ophiostoma* known from the housekeeping genes (Lim *et al.* 2004), suggesting all these genes shared the same evolutionary history.

The occurrence of truncated mating-type genes in opposite *MAT* idiomorphs may suggest a common homothallic ancestor carrying a complete set of *MAT* genes, for instance all *MAT1-1-1*, *MAT1-1-2*, *MAT1-1-3*, and *MAT1-2-1* located at a single locus in the same order as *Sordaria macrospora* [Figure 15.2 in (Debuchy and Turgeon 2006)]. The *MAT1-1* and *MAT1-2* idiomorphs could have arisen from the loss of HMG and α-box domain sequences as a result of multiple translocation breaks and segregations (Paoletti *et al.* 2005b). This scenario had been proposed to explain the *MAT* locus evolution in *Cordyceps takamontana* (Yokoyama *et al.* 2003), members of *Botrytis* and *Sclerotinum* (Amselem *et al.* 2011), as well as the *Aspergilli* (Galagan *et al.* 2005; Paoletti *et al.* 2005b). However, this model of evolution is very unlikely based on the criterion of parsimony. Also *G. clavigera* and related fungi have syntenic order of *MAT* genes and may share a common heterothallic ancestor within the Sordariomycetes as discussed above.

### Deletion of the α-box domain due to evolutionary degeneration of *MAT1-1-1* genes

The deletion/removal of the α-box domain in the truncated *MAT1-1-1* gene may have been selected to avoid universal compatibility or haploid selfing (self-fertility) that involves no sexual recombination in reproduction (Billiard *et al.* 2011, 2012). In fact, the expression of additional α-box domain may interfere or compete with the signal from the “resident/ original” HMG domain at the same locus (Coppin *et al.* 1997), therefore the “additional” *MAT1-1-1* gene may have been “inactivated” for functions in mating (Coppin *et al.* 1997) after the integration into the *MAT* locus as a result of unequal recombination. Under laboratory conditions, artificial association of both mating-type loci in the same nucleus of a heterothallic *Neurospora crassa* isolate led to inhibition of growth and the formation of barren perithecia in crosses with a tester (Perkins 1972). Transgenic dual maters (carrying genes of both mating-types) were also unable to produce progeny in isolates of *N. crassa* (Glass *et al.* 1990), *Podospora anserina* (Coppin and Debuchy 2000) and *Cochliobolus heterostrophus* (Turgeon *et al.* 1993).

In the absence of purifying selection, genes that have lost their original functions accumulate mutations and degenerate to become pseudogenes. Our data illustrated that the truncated *MAT1-1-1* gene in *O. montium* is highly eroded (31% in length compared with its ‘full length’ homolog) and could be a pseudogene. Similarly, the truncated *MAT1-1-1* gene reported in *Cordyceps takamontana* (known as *Isaria tenuipes*) was a pseudogene with accumulated mutations and stop codons (Yokoyama *et al.* 2005). The 3′-end of the *MAT1-1-1* gene in the *MAT1-2* idiomorph of *H. jecorina* was also considered to be nonfunctional because the flanking region contained multiple stop codons with no translational start point detected (Seidl *et al.* 2009). Also, the duplicated homeodomain transcription factors at *A* mating-type locus in *Coprinopsis cinerea* (Basidiomycota) were deleted and inactivated progressively (Kues *et al.* 2012). The *MAT* locus in yeasts was considered a “deletion hotspot” with a continued process of evolutionary deletions, gene truncation and transpositions on chromosomal genes located beside the *MAT* locus after the mating-type switching event (Gordon *et al.* 2011).

The truncated *MAT1-1-1* genes in *G. clavigera* and relatives could have lost their original function in sexual reproduction but the degree of degeneration is not the same as the homolog in *O. montium*. However, these genes do not contain any stop codons and introns. The CODMEL tests indicated purifying selection at the inter-specific level due to accelerated rate of amino acid changes. The deletion of the α-box domain in the truncated *MAT1-1-1* genes also did not prevent its expression as it has already been demonstrated in *Magnaporthe orzyae* (Kanamori *et al.* 2007). It is possible that the truncated genes have evolved new functions through adaptive evolution because the organization has been maintained in the entire phylogenetic clade. In contrast, the 3′-terminal truncated *SXI1α* gene at the *MAT* locus of *Cryptococcus neoformans* serotype-AD hybrid (Basidiomycota) is still functional in sexual reproduction and may even promote cell fusion (Lin *et al.* 2007). Additional in-depth molecular genetics studies and experiments are necessary to elucidate the possible biological functions of the truncated *MAT1-1-1* gene.

### Implications for the mating strategies and breeding systems in fungi

Fungi within the Ophiostomatales have complex mating behavior that range from strict outcrossing (heterothallism) to haploid-selfing (homothallism) (Gorton and Webber 2000; Carlier *et al.* 2006). Also, *Ophiostoma ulmi* had been thought to perform ‘pseudoselfing’ by a process involved in mutation at the *MAT* locus or introgressed *MAT* genes that led to a mating-type switch (Brasier and Gibbs 1975). Our results reflected that most members in *Grosmannia* are heterothallic in genetic makeup and they require a partner (outcrossing) to produce perithecia in life histories. These molecular data are largely congruent to the classical data from pairing-cultures, except that *G. robusta* was reported to produce perithecia readily in culture without pairing cultures (Jacobs and Wingfield 2001). Also evidence of incongruence between molecular data and classical data were reported in *O. quercus*, as *MAT1-2-1* genes appeared to be present in both mating partners that are able to cross (Wilken *et al.* 2012). Previous phylogenetic data demonstrated that homothallism has evolved multiple times independently from within heterothallic ascomycetes (Yun *et al.* 1999; Strandberg *et al.* 2010; Billiard *et al.* 2011). Further characterization of the *MAT* loci from selfing species in Ophiostomatales based on classical mating studies could verify if homothallic members have been derived from a heterothallic ancestor, infer the phylogenetic relationships between the *MAT* locus organizations and reveal the mechanisms underlying the lifestyle changes.

Heterothallic (outcrossing) fungi gain benefits from recombination by increased genetic diversity and repaired mutation (Heitman *et al.* 2007). The presence of both mating-type isolates in *G. clavigera* at different spatial scales is consistent with high levels of genotypic diversity found in this fungus (Tsui *et al.* 2012). The finding of linkage equilibrium among microsatellite markers further indicated that sexual reproduction is a major factor influencing the population genetic structure and epidemiology of *G. clavigera* (Tsui *et al.* 2012). If *G. clavigera* undergoes a sexual cycle regularly, the ascospores and ascomata should be discovered without difficulty. It was proposed that the inability to find the ascomata in nature is due to the inappropriate sampling methodology, or collecting plant material at the wrong stage in the life cycle (Sommerhalder *et al.* 2006). Unfortunately, several attempts to cross complement isolates *in vitro* proved unsuccessful. Other possible explanations may be mutations in genes regulating the sexual development or environmental factors that reduce ascomata formation (Bennett *et al.* 2003).

Finally, our results revealed the presence of homologous *MAT* genes in *Llo*, *Lt*, *Lw*, and *Llun*, which have long been considered to be asexual (Jacobs and Wingfield 2001). We also found 1:1 mating-type ratio in *L. longiclavatum* populations. Evidence of purifying selection on the *MAT* genes at the inter-specific level indicated that sexual reproduction is important in nature or occurs regularly (López-Villavicencio *et al.* 2010). Our data showed that these fungi previously deemed asexual have the potential to reproduce sexually, as was demonstrated for other asexual fungi such as *Penicillium*, *Aspergillus*, and *Fusarium* (Butler 2007; Ropars *et al.* 2012). This ability potentially increases genetic variability and can enhance fitness of fungal pathogens in new, ecological niches (Coppin *et al.* 1997).

## Supplementary Material

Supporting Information
