# Supplementary material for: Unequal Recombination and Evolution of the Mating-Type (MAT) Loci in the Pathogenic Fungus Grosmannia clavigera and Relatives
Source: G3 (Bethesda). 2013 Mar 1;3(3):465–80. doi: 10.1534/g3.112.004986 (PMC3583454; doi:10.1534/g3.112.004986)
Supplement: Supporting Information [file supp_3_3_465__index.html]

Supporting Information 

# Unequal Recombination and Evolution of the Mating-Type (*MAT*) Loci in the Pathogenic Fungus *Grosmannia clavigera* and Relatives

## Supporting Information for Tsui *et al.*, 2013

**Files in this Data Supplement:**

- Supporting Information - Figures S1-S5 and Tables S1-S4 (PDF, 657 KB)
- Figure S1 - Dotplot analyses of mating-type idiomorphs in *L. longiclavatum*, *L. terebrantis*, *G. aurea*, *G. huntii*, and *L. lundbergii* (PDF, 90 KB)
- Figure S2 - Homology between the *MAT* loci of *O. montium* and *O. novo-ulmi* subsp. *novo-ulmi* (PDF, 156 KB)
- Figure S3 - A distance tree generated from MEGA showing the phylogenetic relationships among ascomycetes inferred from the α-box domain of the MAT1-1-1 (75 amino acid characters) (PDF, 190 KB)
- Figure S4 - Gene genealogies of the *MAT1-1-2* (1045 characters a maximum parsimony tree), *MAT1-1-3* (574 characters, a neighbor-joining tree), and *MAT1-2-1* (858 characters, a neighbor-joining tree) demonstrating the phylogenetic relationships among *G. clavigera* and related species (PDF, 126 KB)
- Figure S5 - Gene genealogies of the nucleotide sequences of (A) *SLA* (898 characters, a maximum parsimony tree), (B) *COX13* (788 characters, a neighbor-joining tree), and (C) *APN* (1444 characters, neighbor-joining tree) genes demonstrating the phylogenetic relationships among *G. clavigera* and related species (PDF, 289 KB)
- Table S2 - Pair-wise nucleotide similarities among *MAT* idiomorphs of different isolates and species (PDF, 101 KB)
- Table S3 - Parameter estimates and likelihood values of the various models of codon evolution using CODEML in PAML (PDF, 113 KB)
- Table S4 - Gene expression level of *MAT1-2-1* and truncated *MAT1-1-1* genes, as well as other major flanking genes along the same chromosome in 12hr control and terpene treatment conditions (PDF, 88 KB)
- Table S1 - List of sequencing primers used in primer walking (.xls, 67 KB)
