# Supplementary material for: Unequal Recombination and Evolution of the Mating-Type (MAT) Loci in the Pathogenic Fungus Grosmannia clavigera and Relatives
Source: G3 (Bethesda). 2013 Mar 1;3(3):465–80. doi: 10.1534/g3.112.004986 (PMC3583454; doi:10.1534/g3.112.004986)
Supplement: Supporting Information [file supp_3.3.465_004986SI.pdf]

G3·Genes | Genomes | Genetics

Title: Unequal recombination and evolution of the mating-type (*MAT*) loci in the pathogenic fungus *Grosmannia clavigera* and relatives

Clement K.-M. Tsui,[*,1] Scott DiGuistini,[§] Ye Wang,[§] Nicolas Feau,[*] Braham Dhillon,[*] Jörg Bohlmann,[*,±] and Richard C. Hamelin[*,+]

[*]Department of Forest Sciences, The University of British Columbia, Vancouver, BC, Canada V6T 1Z4
[§]Department of Wood Science, The University of British Columbia, Vancouver, BC, Canada V6T 1Z4
[±]Michael Smith Laboratory, The University of British Columbia, Vancouver, BC, Canada V6T 1Z4
[+]Natural Resources Canada, Laurentian Forestry Centre, 1055 du P.E.P.S., Québec, QC, Canada G1V 4C7

DOI: 10.1534/g3.112.004986

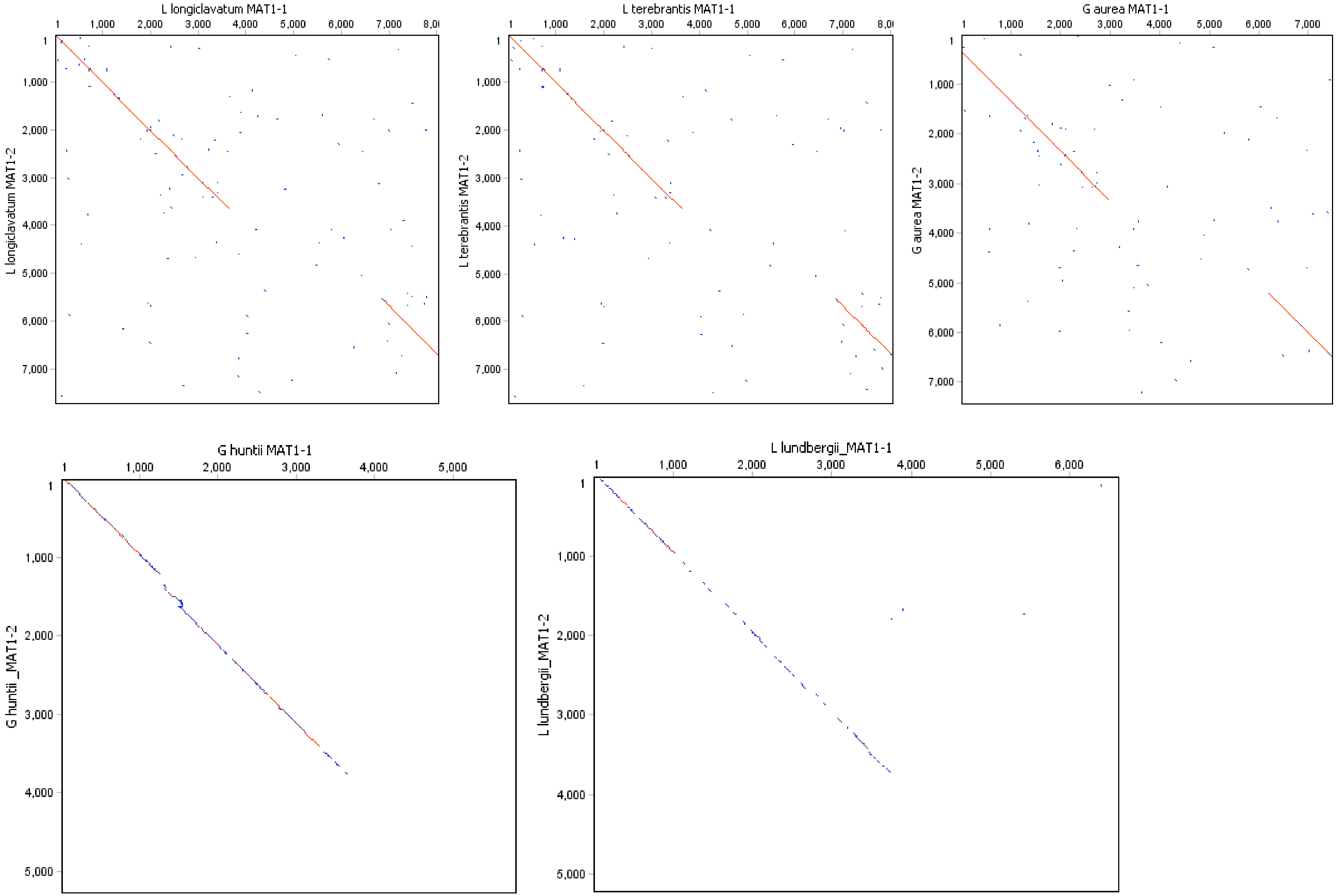

Figure S1 Dotplot analyses of mating-type idiomorphs in *L. longiclavatum, L. terebrantis*, *G. aurea*, *G. huntii,* and *L. lundbergii.*

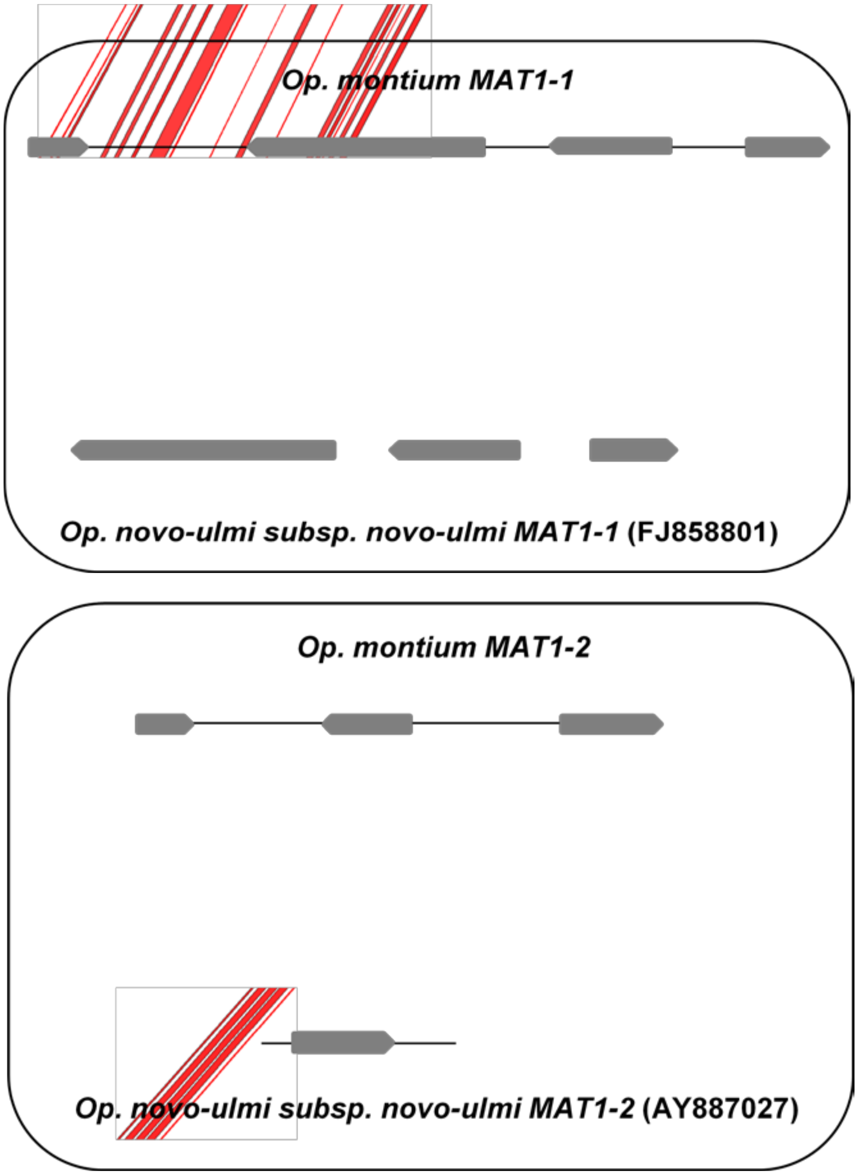

Figure S2 Homology between the *MAT* loci of *O. montium* and *O. novo-ulmi* subsp*. novo-ulmi*. The diagram was prepared from the output of Artemis Comparison Tool.

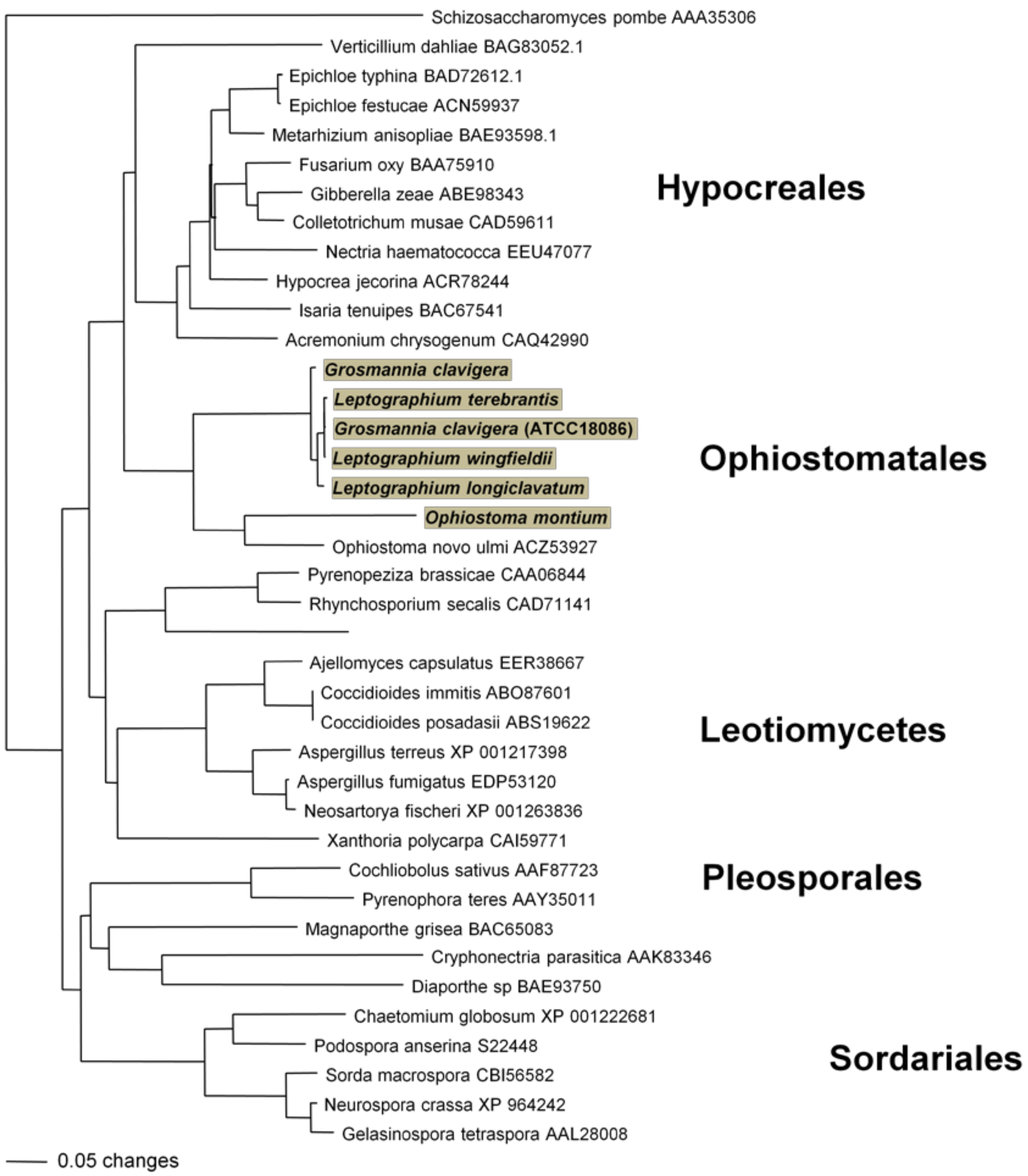

Figure S3 A distance tree generated from MEGA showing the phylogenetic relationships among ascomycetes inferred from the α-box domain of the MAT1-1-1 (75 amino acid characters). Number on branches indicated bootstrap support over 60% (1000 pseudoreplicates) from neighbor-joining algorithm.

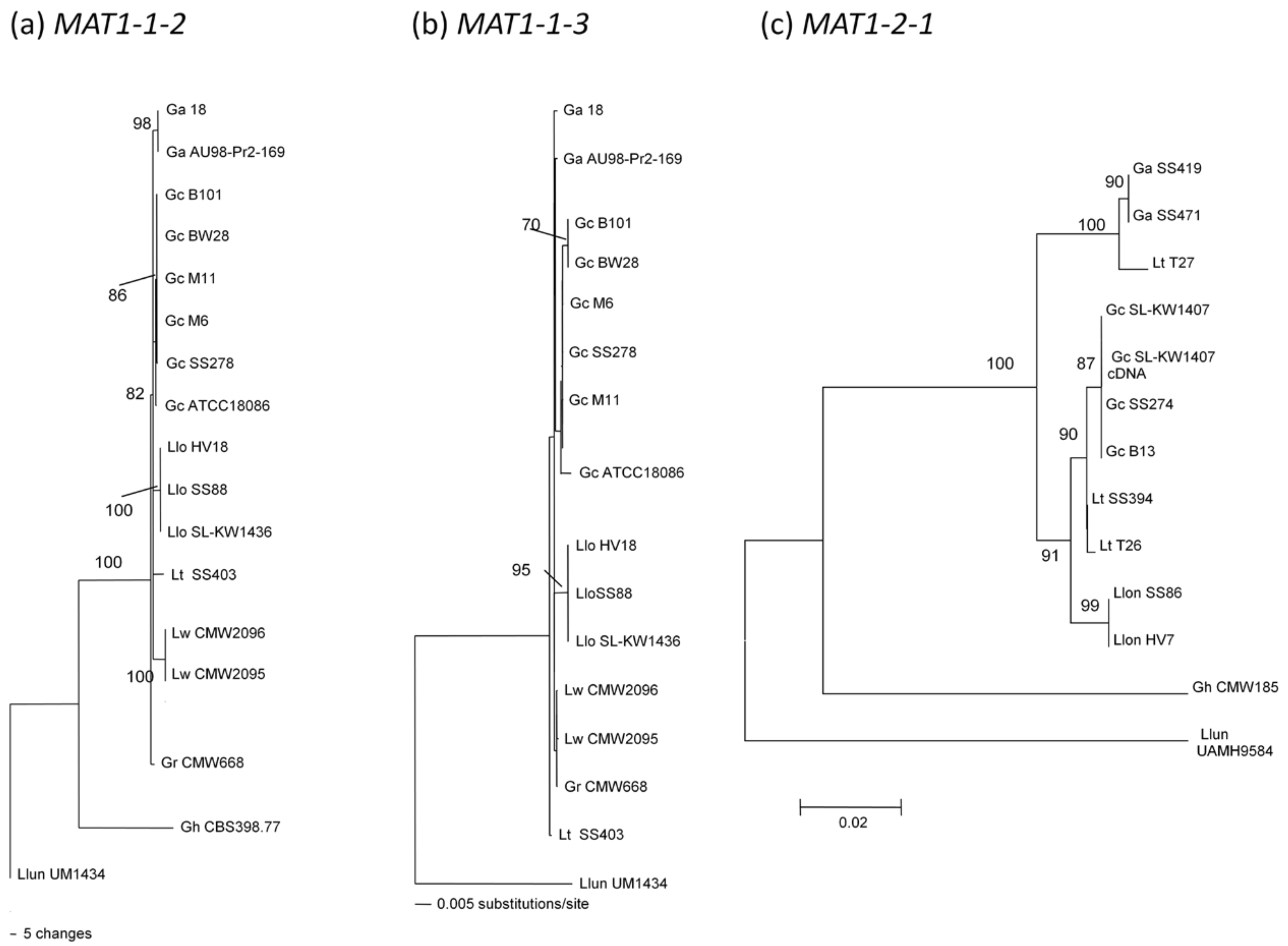

Figure S4 Gene genealogies of the *MAT1-1-2* (1045 characters a maximum parsimony tree), *MAT1-1-3* (574 characters, a neighbor-joining tree), and *MAT1-2-1* (858 characters, a neighbor-joining tree) demonstrating the phylogenetic relationships among *G. clavigera* and related species.

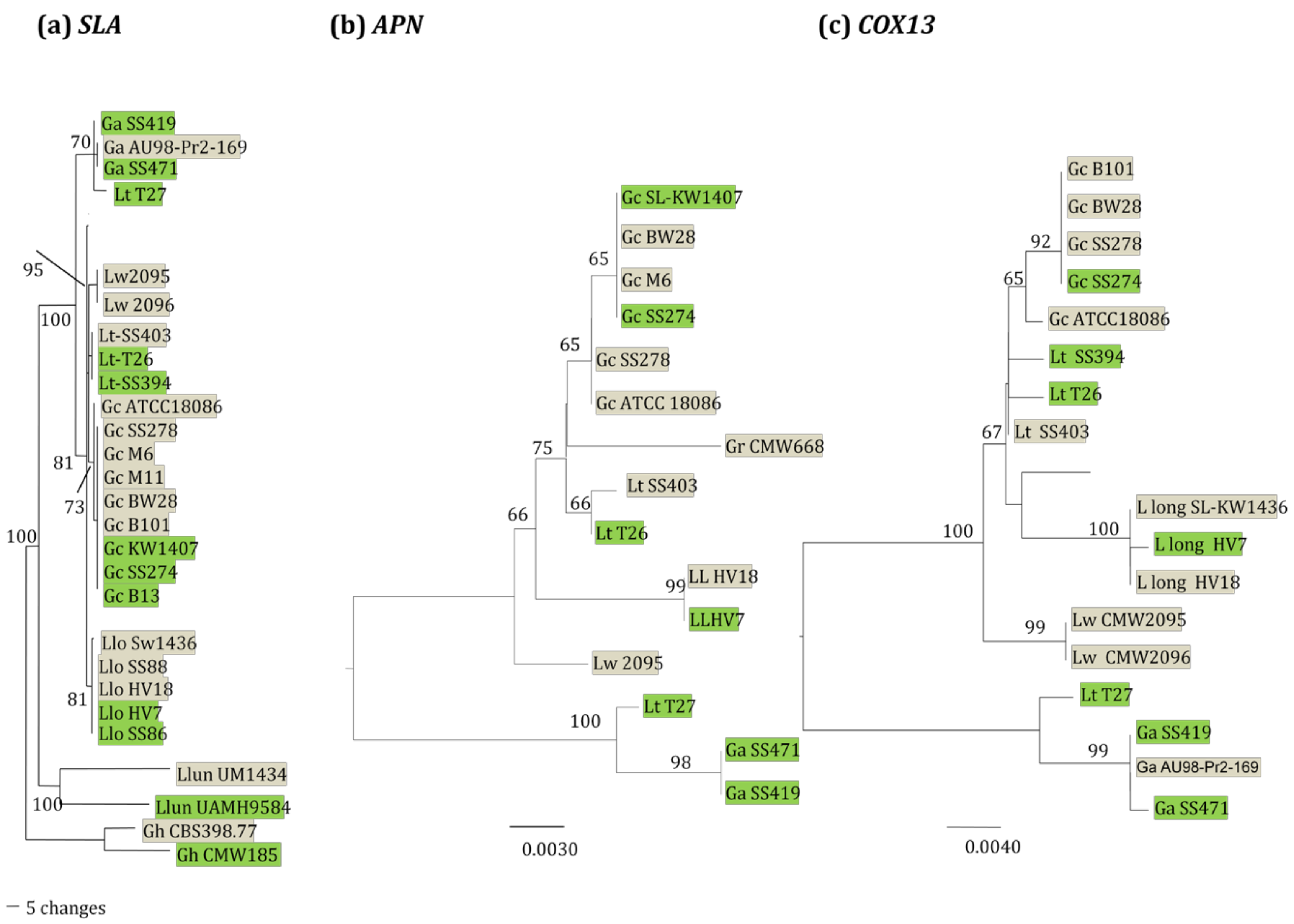

Figure S5 Gene genealogies of the nucleotide sequences of (A) *SLA* (898 characters, a maximum parsimony tree), (B) *COX13* (788 characters, a neighbor-joining tree), and (C) *APN* (1444 characters, neighbor-joining tree) genes demonstrating the phylogenetic relationships among *G. clavigera* and related species. *MAT1-2* isolates are indicated in green while *MAT1-1* isolates are highlighted in grey.

Table S1   List of sequencing primers used in primer walking

Table S1 is available for download at http://www.g3journal.org/lookup/suppl/doi:10.1534/g3.112.004986/-/DC1.

Table S2 Pair-wise nucleotide similarities among *MAT* idiomorphs of different isolates and species.

a. Percentage nucleotide similarity among *MAT1-1* idiomorphs

		1	2	3	4	5	6	7	8	9	10	11	12	13	14	15	16	17
**1**	**L lung UM1434**																	
**2**	**Gh CBS398.77**	77.6																
**3**	**Gc M6**	76.4	75.4															
**4**	**Gc M11**	76.5	75.3	99.8														
**5**	**Gc SS278**	76.4	75.2	99.9	99.8													
**6**	**Gc BW28**	76.4	75.3	99.9	99.8	100												
**7**	**Gc B101**	76.4	75.3	99.9	99.8	100	100											
**8**	**Gc ATCC 18086**	76.2	75.1	98.9	98.8	99	99	99										
**9**	**Lw CMW 2096**	76.3	74.9	98.1	98.1	98.2	98.7	98.7	98.5									
**10**	**Lw CMW 2095**	76.4	75.1	98.6	98.6	98.7	98.7	98.7	98.5	99.9								
**11**	**Ga OA18_A27**	78.4	76.6	86.8	86.8	86.9	97.1	97.1	97.2	86.8	97.1							
**12**	**Ga AU98-Pr2**	73.8	72.7	87.5	87.4	87.5	96.1	96.1	95.8	87.5	96.1	99.9						
**13**	**L lo SS88**	76.1	74.8	97.7	97.7	97.8	97.9	97.9	97.7	97.7	98	96.2	95.1					
**14**	**L lo SL-KW1436**	76	74.7	97.8	97.8	97.9	97.9	97.9	97.6	97.8	97.9	96.6	95.4	100				
**15**	**L lo HV18**	76.1	74.9	97.9	97.8	97.9	97.9	97.9	97.7	97.9	98	96.7	95.4	99.9	99.9			
**16**	**Lt SS403**	76.5	75.2	98.6	98.6	98.7	98.9	98.9	98.6	98.8	99	96.8	96	97.9	98	98.1		
**17**	**Gr CMW668**	76.7	75.2	98.2	98.2	98.3	98.3	98.3	97.9	98.1	98.2	97.1	95.5	97.8	97.7	97.8	98.4	

b. Percentage nucleotide similarity among *MAT1-2* idiomorphs

		1	2	3	4	5	6	7	8	9	10	11	12
**1**	**Llun UAMH9584**												
**2**	**Gh CMW185**	71.9											
**3**	**Gc Sl-KW1407**	74	72.9										
**4**	**Gc SS274**	74	72.9	100									
**5**	**Gc B13**	74	72.9	99.9	99.9								
**6**	**Ga SS419**	72.8	71.4	94.1	92.5	92.5							
**7**	**Ga SS 471**	73.3	72.1	94.3	94.2	94.2	99.8						
**8**	**Llo SS86**	73.6	72.5	97.9	97.1	97.1	92.2	93.6					
**9**	**Llo HV7**	73.7	72.5	98	97.9	97.8	93.4	93.6	99.9				
**10**	**Lt SS394**	74.1	72.9	99.1	98.5	98.5	92.9	94.5	97.6	98.2			
**11**	**Lt T26**	74	72.8	98.9	98.9	98.8	94.2	94.4	98.1	98.1	99.8		
**12**	**Lt T27**	71.1	69.9	94.2	91.9	91.8	97	98.4	91.5	93.4	92.4	94.2	

Table S3 Parameter estimates and likelihood values of the various models of codon evolution using CODEML in PAML

Test of selection on *MAT* genes among different species

	**Model**	**-lnL**	**Models comparison**	**2ΔL**	***Pr.***
**Full length *MAT1-1-1***	M1a (neutral)	2756.64	M1a vs. M2a	2.41	ns
	M2a (selection)	2755.43			
	M7 (beta)	2756.82			
	M8 (beta + ω)	2755.44	M7 vs. M8	2.77	ns
**Truncated *MAT1-1-1***	M1a (neutral)	2636.82	M1a vs. M2a	0.0	ns
	M2a (selection)	2636.82			
	M7 (beta)	2636.80	M7 vs. M8	0.0	ns
	M8 (beta + ω)	2636.80			
MAT1-2-1	M1a (neutral)	1373.38	M1a vs. M2a	0.95	ns
	M2a (selection)	1372.90			
	M7 (beta)	1373.42	M7 vs. M8		ns
	M8 (beta + ω)	1372.90			

⇨ **Nothing significant**

Table S4 Gene expression level of *MAT1-2-1* and truncated *MAT1-1-1* genes, as well as other major flanking genes along the same chromosome in 12hr control and terpene treatment conditions. Data extracted from DiGuistini *et al.* (2011).

				**12hr simple control**				**12hr terpene treatment**			
**Seq. Name**	**CMQ_ #**	**Seq. length**	**Seq. Description**	**Expression values**	**Normalized expression values**	**Total gene reads**	**RPKM**	**Expression values**	**Normalized expression values**	**Total gene reads**	**RPKM**
**GLEAN_5479**	5197	3108	cytoskeleton assembly control protein	58.14	45.9	271	58.14	41.09	42.3	49	41.09
**GLEAN_848**	5309	1596	Predicted protein/truncated MAT1-1-1	8.77	6.9	21	8.77	3.27	3.4	2	3.27
**GLEAN_5480**	5376	858	mating type protein 1-2-1	17.1	13.5	22	17.1	3.04	3.1	1	3.04
**GLEAN_847**	5208	1065	Predicted protein	0	0	0	0	0	0	0	0
**GLEAN_5481**	5136	1392	Predicted protein	3.83	3	8	3.83	3.74	3.9	2	3.74
**GLEAN_845**	5282	375	cytochrome c oxidase subunit	686.32	541.7	386	686.32	688.03	708.4	99	688.03
**GLEAN_5482**	5213	1761	PA-domain protein	45.06	35.6	119	45.06	10.36	10.7	7	10.36
**GLEAN_844**	5212	2388	DNA lyase	34.9	27.5	125	34.9	22.92	23.6	21	22.92
