# Supplementary material for: Unequal Recombination and Evolution of the Mating-Type (MAT) Loci in the Pathogenic Fungus Grosmannia clavigera and Relatives
Source: G3 (Bethesda). 2013 Mar 1;3(3):465–80. doi: 10.1534/g3.112.004986 (PMC3583454; doi:10.1534/g3.112.004986)
Supplement: Supporting Information [file supp_3.3.465_FigureS1.pdf]

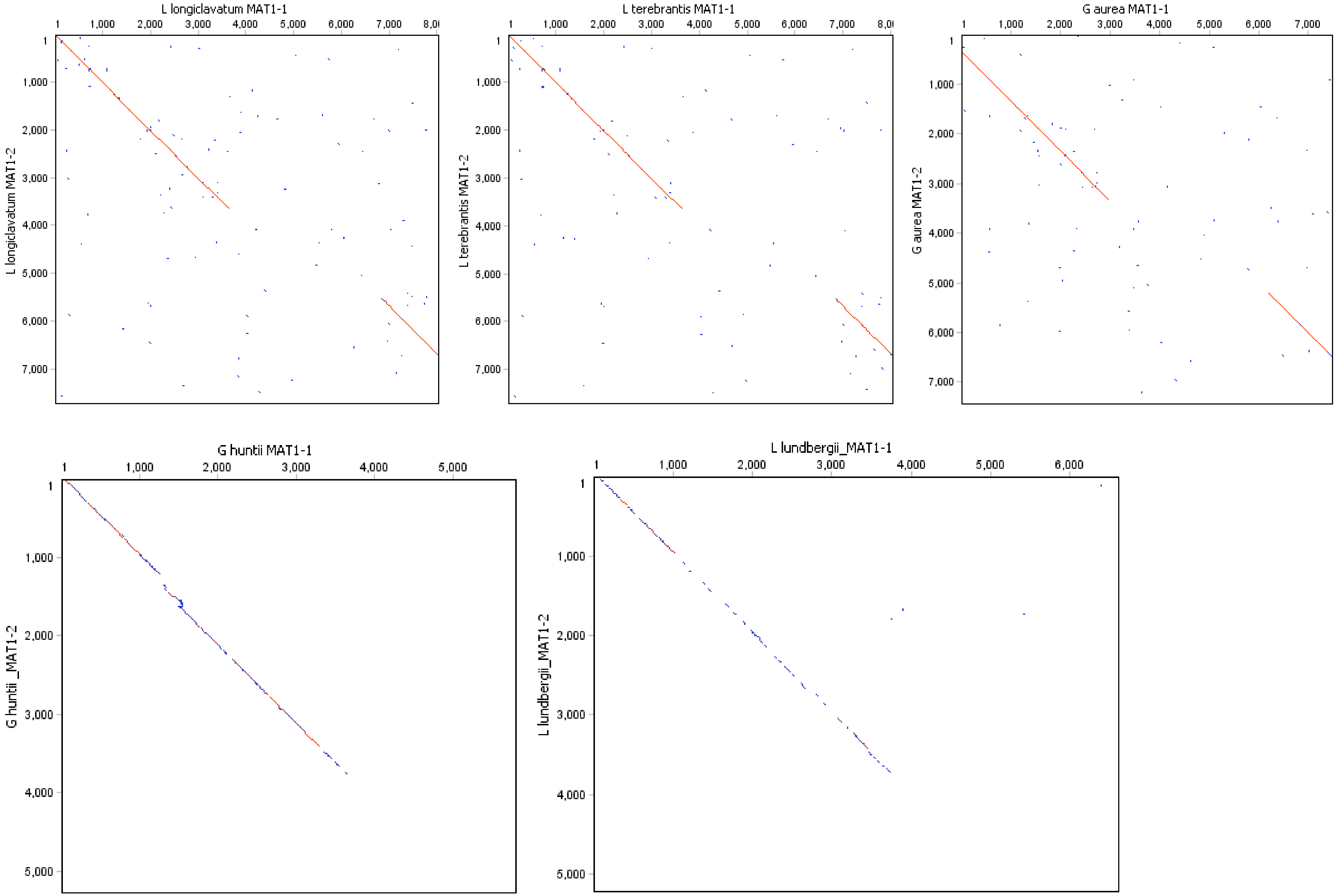

Figure S1 Dotplot analyses of mating-type idiomorphs in *L. longiclavatum, L. terebrantis*, *G. aurea*, *G. huntii,* and *L. lundbergii.*
