# Supplementary material for: Unequal Recombination and Evolution of the Mating-Type (MAT) Loci in the Pathogenic Fungus Grosmannia clavigera and Relatives
Source: G3 (Bethesda). 2013 Mar 1;3(3):465–80. doi: 10.1534/g3.112.004986 (PMC3583454; doi:10.1534/g3.112.004986)
Supplement: Supporting Information [file supp_3.3.465_FigureS2.pdf]

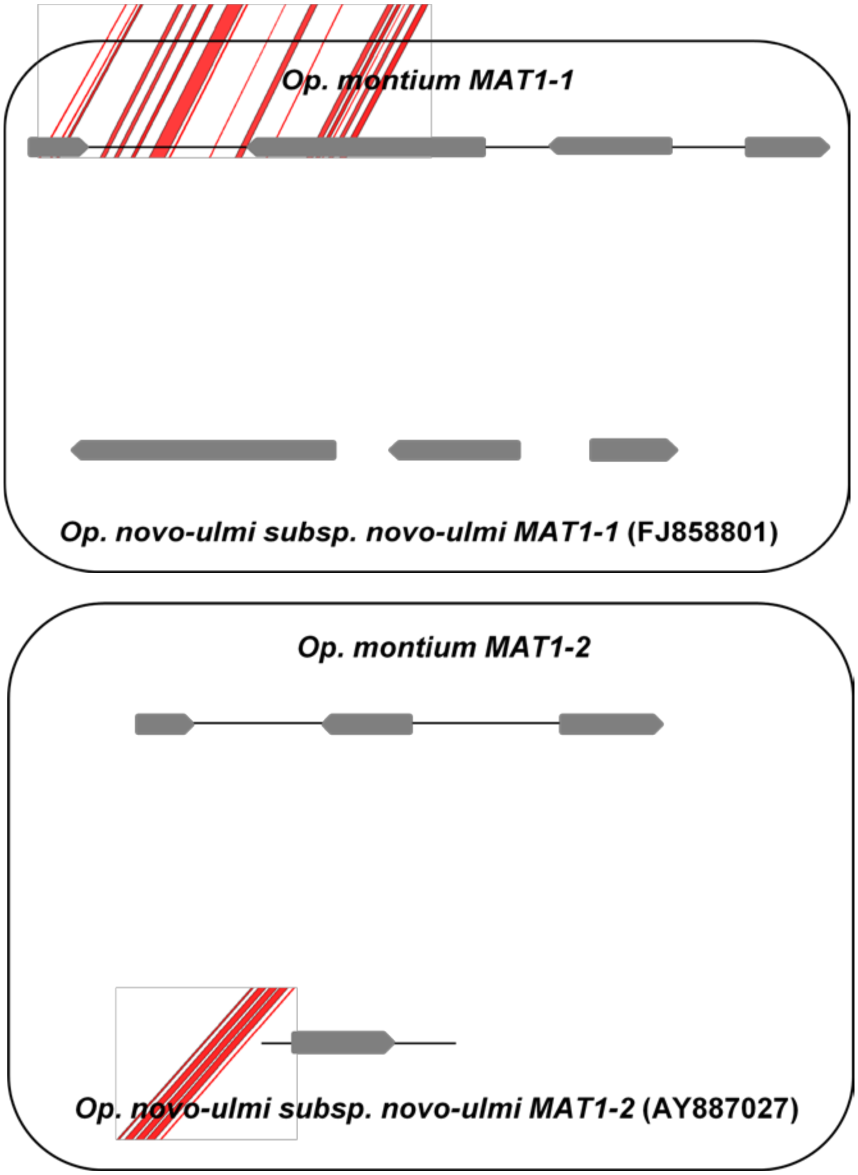

Figure S2 Homology between the *MAT* loci of *O. montium* and *O. novo-ulmi* subsp. *novo-ulmi*. The diagram was prepared from the output of Artemis Comparison Tool.
