# Supplementary material for: Unequal Recombination and Evolution of the Mating-Type (MAT) Loci in the Pathogenic Fungus Grosmannia clavigera and Relatives
Source: G3 (Bethesda). 2013 Mar 1;3(3):465–80. doi: 10.1534/g3.112.004986 (PMC3583454; doi:10.1534/g3.112.004986)
Supplement: Supporting Information [file supp_3.3.465_FigureS3.pdf]

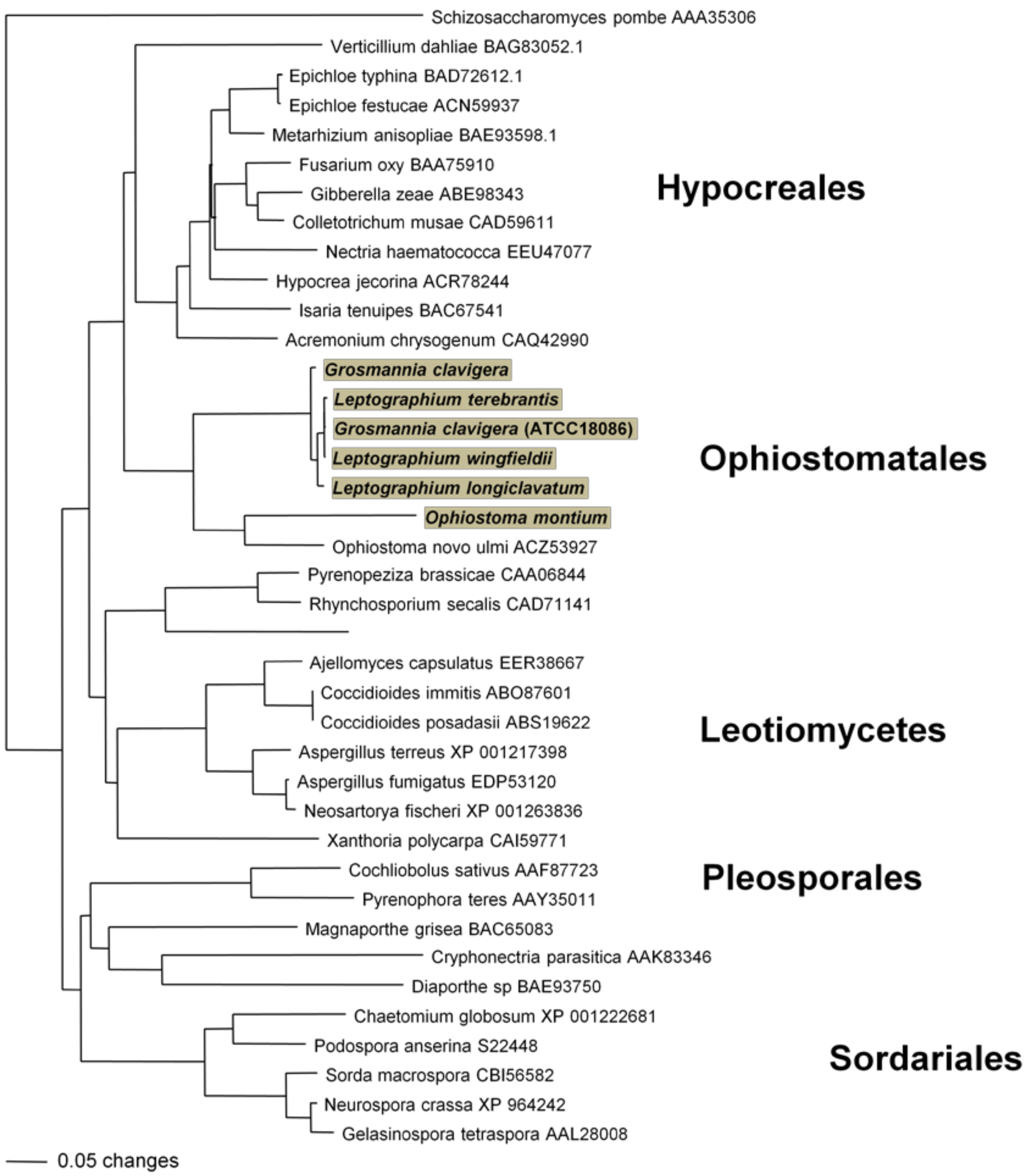

Figure S3 A distance tree generated from MEGA showing the phylogenetic relationships among ascomycetes inferred from the α-box domain of the MAT1-1-1 (75 amino acid characters). Number on branches indicated bootstrap support over 60% (1000 pseudoreplicates) from neighbor-joining algorithm.
