# Supplementary material for: Unequal Recombination and Evolution of the Mating-Type (MAT) Loci in the Pathogenic Fungus Grosmannia clavigera and Relatives
Source: G3 (Bethesda). 2013 Mar 1;3(3):465–80. doi: 10.1534/g3.112.004986 (PMC3583454; doi:10.1534/g3.112.004986)
Supplement: Supporting Information [file supp_3.3.465_FigureS4.pdf]

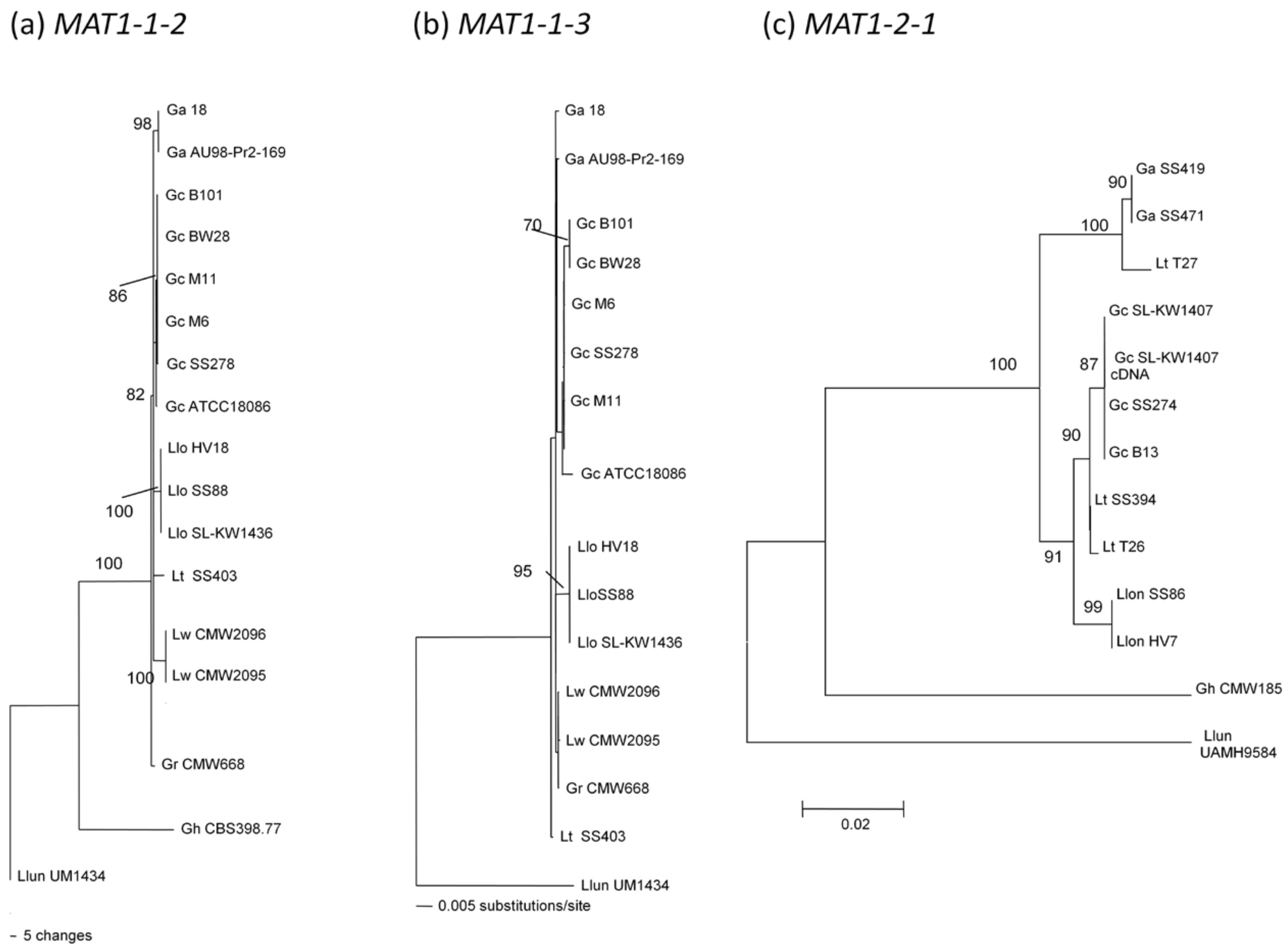


**Figure S4** Gene genealogies of the *MAT1-1-2* (1045 characters a maximum parsimony tree), *MAT1-1-3* (574 characters, a neighbor-joining tree), and *MAT1-2-1* (858 characters, a neighbor-joining tree) demonstrating the phylogenetic relationships among *G. clavigera* and related species.
