# Supplementary material for: Unequal Recombination and Evolution of the Mating-Type (MAT) Loci in the Pathogenic Fungus Grosmannia clavigera and Relatives
Source: G3 (Bethesda). 2013 Mar 1;3(3):465–80. doi: 10.1534/g3.112.004986 (PMC3583454; doi:10.1534/g3.112.004986)
Supplement: Supporting Information [file supp_3.3.465_FigureS5.pdf]

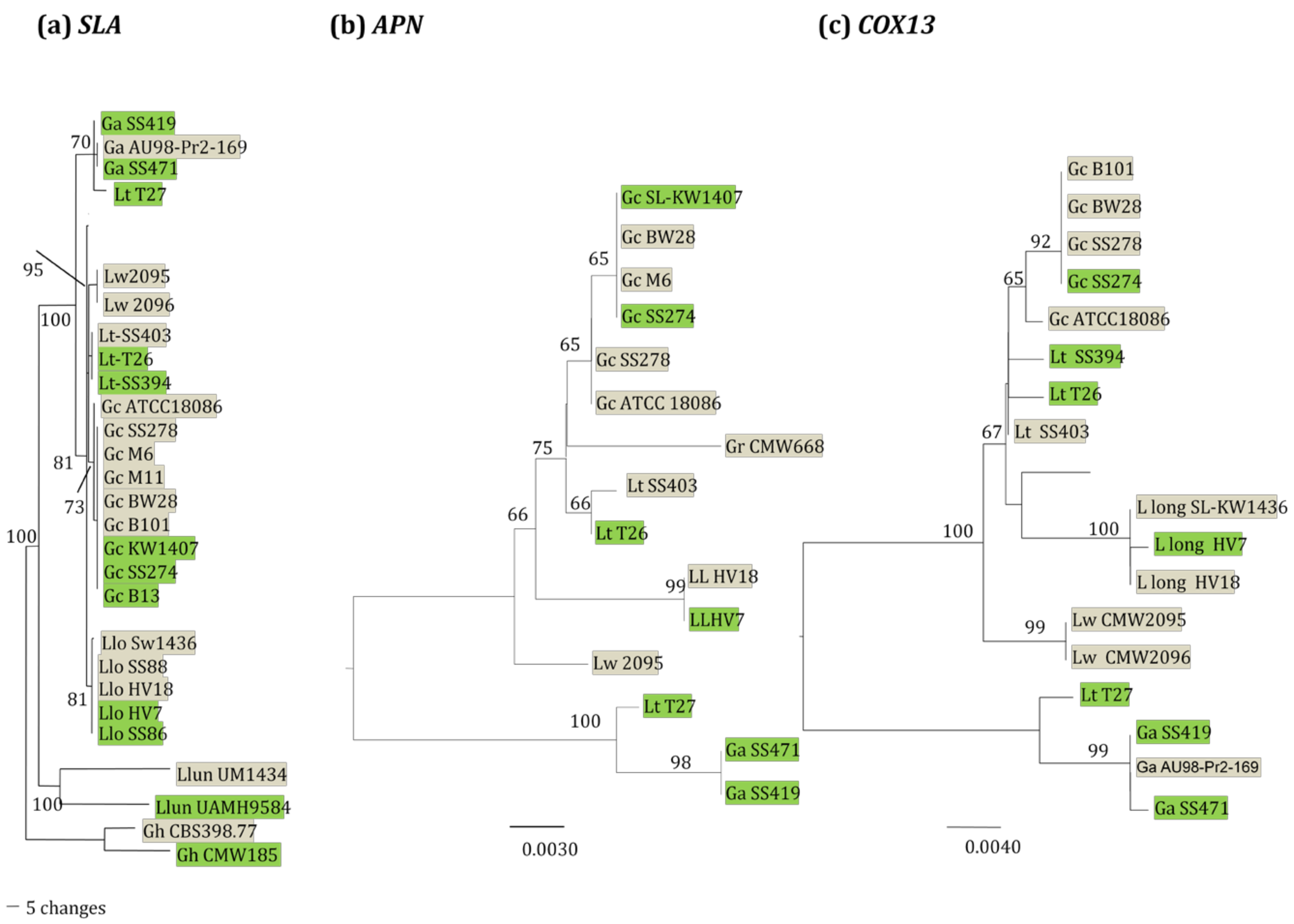

Figure S5 Gene genealogies of the nucleotide sequences of (A) *SLA* (898 characters, a maximum parsimony tree), (B) *COX13* (788 characters, a neighbor-joining tree), and (C) *APN* (1444 characters, neighbor-joining tree) genes demonstrating the phylogenetic relationships among *G. clavigera* and related species. *MAT1-2* isolates are indicated in green while *MAT1-1* isolates are highlighted in grey.
