# Supplementary material for: Unequal Recombination and Evolution of the Mating-Type (MAT) Loci in the Pathogenic Fungus Grosmannia clavigera and Relatives
Source: G3 (Bethesda). 2013 Mar 1;3(3):465–80. doi: 10.1534/g3.112.004986 (PMC3583454; doi:10.1534/g3.112.004986)
Supplement: Supporting Information [file supp_3.3.465_TableS2.pdf]

Table S2 Pair-wise nucleotide similarities among *MAT* idiomorphs of different isolates and species.

a. Percentage nucleotide similarity among *MAT1-1* idiomorphs

		1	2	3	4	5	6	7	8	9	10	11	12	13	14	15	16	17
**1**	**L lung UM1434**																	
**2**	**Gh CBS398.77**	77.6																
**3**	**Gc M6**	76.4	75.4															
**4**	**Gc M11**	76.5	75.3	99.8														
**5**	**Gc SS278**	76.4	75.2	99.9	99.8													
**6**	**Gc BW28**	76.4	75.3	99.9	99.8	100												
**7**	**Gc B101**	76.4	75.3	99.9	99.8	100	100											
**8**	**Gc ATCC 18086**	76.2	75.1	98.9	98.8	99	99	99										
**9**	**Lw CMW 2096**	76.3	74.9	98.1	98.1	98.2	98.7	98.7	98.5									
**10**	**Lw CMW 2095**	76.4	75.1	98.6	98.6	98.7	98.7	98.7	98.5	99.9								
**11**	**Ga OA18_A27**	78.4	76.6	86.8	86.8	86.9	97.1	97.1	97.2	86.8	97.1							
**12**	**Ga AU98-Pr2**	73.8	72.7	87.5	87.4	87.5	96.1	96.1	95.8	87.5	96.1	99.9						
**13**	**L lo SS88**	76.1	74.8	97.7	97.7	97.8	97.9	97.9	97.7	97.7	98	96.2	95.1					
**14**	**L lo SL-KW1436**	76	74.7	97.8	97.8	97.9	97.9	97.9	97.6	97.8	97.9	96.6	95.4	100				
**15**	**L lo HV18**	76.1	74.9	97.9	97.8	97.9	97.9	97.9	97.7	97.9	98	96.7	95.4	99.9	99.9			
**16**	**Lt SS403**	76.5	75.2	98.6	98.6	98.7	98.9	98.9	98.6	98.8	99	96.8	96	97.9	98	98.1		
**17**	**Gr CMW668**	76.7	75.2	98.2	98.2	98.3	98.3	98.3	97.9	98.1	98.2	97.1	95.5	97.8	97.7	97.8	98.4	

b. Percentage nucleotide similarity among *MAT1-2* idiomorphs

		1	2	3	4	5	6	7	8	9	10	11	12
**1**	**Llun UAMH9584**												
**2**	**Gh CMW185**	71.9											
**3**	**Gc Sl-KW1407**	74	72.9										
**4**	**Gc SS274**	74	72.9	100									
**5**	**Gc B13**	74	72.9	99.9	99.9								
**6**	**Ga SS419**	72.8	71.4	94.1	92.5	92.5							
**7**	**Ga SS 471**	73.3	72.1	94.3	94.2	94.2	99.8						
**8**	**Llo SS86**	73.6	72.5	97.9	97.1	97.1	92.2	93.6					
**9**	**Llo HV7**	73.7	72.5	98	97.9	97.8	93.4	93.6	99.9				
**10**	**Lt SS394**	74.1	72.9	99.1	98.5	98.5	92.9	94.5	97.6	98.2			
**11**	**Lt T26**	74	72.8	98.9	98.9	98.8	94.2	94.4	98.1	98.1	99.8		
**12**	**Lt T27**	71.1	69.9	94.2	91.9	91.8	97	98.4	91.5	93.4	92.4	94.2	
