# Supplementary material for: Unequal Recombination and Evolution of the Mating-Type (MAT) Loci in the Pathogenic Fungus Grosmannia clavigera and Relatives
Source: G3 (Bethesda). 2013 Mar 1;3(3):465–80. doi: 10.1534/g3.112.004986 (PMC3583454; doi:10.1534/g3.112.004986)
Supplement: Supporting Information [file supp_3.3.465_TableS3.pdf]

Table S3 Parameter estimates and likelihood values of the various models of codon evolution using CODEML in PAML

Test of selection on *MAT* genes among different species

	Model	-lnL	Models comparison	2ΔL	*Pr.*
Full length *MAT1-1-1*	M1a (neutral)	2756.64	M1a vs. M2a	2.41	ns
	M2a (selection)	2755.43			
	M7 (beta)	2756.82			
	M8 (beta + ω)	2755.44	M7 vs. M8	2.77	ns
Truncated *MAT1-1-1*	M1a (neutral)	2636.82	M1a vs. M2a	0.0	ns
	M2a (selection)	2636.82			
	M7 (beta)	2636.80	M7 vs. M8	0.0	ns
	M8 (beta + ω)	2636.80			
MAT1-2-1	M1a (neutral)	1373.38	M1a vs. M2a	0.95	ns
	M2a (selection)	1372.90			
	M7 (beta)	1373.42	M7 vs. M8		ns
	M8 (beta + ω)	1372.90			

⇨ **Nothing significant**
