# Supplementary material for: Unequal Recombination and Evolution of the Mating-Type (MAT) Loci in the Pathogenic Fungus Grosmannia clavigera and Relatives
Source: G3 (Bethesda). 2013 Mar 1;3(3):465–80. doi: 10.1534/g3.112.004986 (PMC3583454; doi:10.1534/g3.112.004986)
Supplement: Supporting Information [file supp_3.3.465_TableS4.pdf]

**Table S4 Gene expression level of *MAT1-2-1* and truncated *MAT1-1-1* genes, as well as other major flanking genes along the same chromosome in 12hr control and terpene treatment conditions.** Data extracted from DiGuistini *et al.* (2011).

| | | | | **12hr simple control** | | | | **12hr terpene treatment** | | | |
|---|---|---|---|---|---|---|---|---|---|---|---|
| **Seq. Name** | **CMQ_ #** | **Seq. length** | **Seq. Description** | **Expression values** | **Normalized expression values** | **Total gene reads** | **RPKM** | **Expression values** | **Normalized expression values** | **Total gene reads** | **RPKM** |
| **GLEAN_5479** | 5197 | 3108 | cytoskeleton assembly control protein | 58.14 | 45.9 | 271 | 58.14 | 41.09 | 42.3 | 49 | 41.09 |
| **GLEAN_848** | 5309 | 1596 | Predicted protein/truncated MAT1-1-1 | 8.77 | 6.9 | 21 | 8.77 | 3.27 | 3.4 | 2 | 3.27 |
| **GLEAN_5480** | 5376 | 858 | mating type protein 1-2-1 | 17.1 | 13.5 | 22 | 17.1 | 3.04 | 3.1 | 1 | 3.04 |
| **GLEAN_847** | 5208 | 1065 | Predicted protein | 0 | 0 | 0 | 0 | 0 | 0 | 0 | 0 |
| **GLEAN_5481** | 5136 | 1392 | Predicted protein | 3.83 | 3 | 8 | 3.83 | 3.74 | 3.9 | 2 | 3.74 |
| **GLEAN_845** | 5282 | 375 | cytochrome c oxidase subunit | 686.32 | 541.7 | 386 | 686.32 | 688.03 | 708.4 | 99 | 688.03 |
| **GLEAN_5482** | 5213 | 1761 | PA-domain protein | 45.06 | 35.6 | 119 | 45.06 | 10.36 | 10.7 | 7 | 10.36 |
| **GLEAN_844** | 5212 | 2388 | DNA lyase | 34.9 | 27.5 | 125 | 34.9 | 22.92 | 23.6 | 21 | 22.92 |
